# Supplementary material for: The proportion of Alzheimer’s disease attributable to apolipoprotein E
Source: NPJ Dement. 2026 Jan 9;2(1):1. doi: 10.1038/s44400-025-00045-9 (PMC12789039; doi:10.1038/s44400-025-00045-9)
Supplement: Supplementary file 2 — Supplementary Information [file 44400_2025_45_MOESM2_ESM.docx]

| **Supplemental table 4. All primary association and PAF calculations across UKB, FinnGen, A4 and ADGC** | | | | | |  |
| --- | --- | --- | --- | --- | --- | --- |
|  |  |  |  |  |  |  |
| Study | Outcome | Exposure | Cases per genotype | Case fraction | RR (95% CI) | PAF (95% CI), % |
| UKB | AD | e2/e2 | 5 | 0.001 | Ref. |  |
|  |  | e2/e3 | 170 | 0.050 | 1.68 (0.69, 4.07) | 2.0 (-2.2, 3.8) |
|  |  | e3/e3 | 1061 | 0.314 | 2.19 (0.91, 5.25) | 17.0 (-3.1, 25.4) |
|  |  | e2/e4 | 81 | 0.024 | 4.03 (1.64, 9.91) | 1.8 (0.9, 2.2) |
|  |  | e3/e4 | 1608 | 0.476 | 8.39 (3.50, 20.13) | 41.9 (34.0, 45.2) |
|  |  | e4/e4 | 456 | 0.135 | 23.36 (9.71, 56.19) | 12.9 (12.1, 13.2) |
|  |  | total | 3381 |  |  | 75.7 (41.7, 89.8) |
|  | All-cause dementia | e2/e2 | 25 | 0.003 | Ref. |  |
|  |  | e2/e3 | 508 | 0.068 | 1.00 (0.67, 1.48) | 0.0 (-3.3, 2.2) |
|  |  | e3/e3 | 2893 | 0.389 | 1.19 (0.81, 1.75) | 6.2 (-9.3, 16.7) |
|  |  | e2/e4 | 214 | 0.029 | 2.12 (1.41, 3.18) | 1.5 (0.8, 2.0) |
|  |  | e3/e4 | 3026 | 0.407 | 3.15 (2.14, 4.64) | 27.8 (21.7, 31.9) |
|  |  | e4/e4 | 764 | 0.103 | 7.71 (5.22, 11.39) | 8.9 (8.3, 9.4) |
|  |  | total | 7430 |  |  | 44.4 (18.2, 62.2) |
| FinnGen | AD | e2/e2 | 12 | 0.001 | Ref. |  |
|  |  | e2/e3 | 512 | 0.043 | 1.63 (1.00, 2.63) | 1.6 (0.0, 2.6) |
|  |  | e3/e3 | 4942 | 0.400 | 2.29 (1.40, 3.71) | 22.7 (11.5, 29.4) |
|  |  | e2/e4 | 297 | 0.025 | 4.44 (2.87, 6.79) | 1.9 (1.6, 2.1) |
|  |  | e3/e4 | 5186 | 0.421 | 6.37 (4.32, 9.25) | 35.6 (32.5, 37.7) |
|  |  | e4/e4 | 1327 | 0.110 | 9.79 (7.44, 12.73) | 9.7 (9.4, 10.0) |
|  |  | total | 12276 |  |  | 71.5 (54.9, 81.7) |
|  | All-cause dementia | e2/e2 | 53 | 0.002 | Ref. |  |
|  |  | e2/e3 | 1558 | 0.055 | 1.11 (0.84, 1.45) | 0.5 (-1.0, 1.7) |
|  |  | e3/e3 | 13051 | 0.457 | 1.33 (1.03, 1.69) | 11.3 (1.5, 18.7) |
|  |  | e2/e4 | 686 | 0.024 | 2.69 (2.08, 3.43) | 1.5 (1.2, 1.7) |
|  |  | e3/e4 | 10910 | 0.382 | 3.00 (2.41, 3.70) | 25.5 (22.3, 27.9) |
|  |  | e4/e4 | 2294 | 0.080 | 6.36 (5.50, 7.25) | 6.8 (6.6, 6.9) |
|  |  | total | 28552 |  |  | 45.6 (30.6, 56.9) |
| A4 | AB+ | e2/e2 | 1 | 0.001 | Ref. |  |
|  |  | e2/e3 | 60 | 0.050 | 3.30 (0.48, 13.21) | 3.5 (-5.1, 4.6) |
|  |  | e3/e3 | 409 | 0.338 | 4.27 (0.64, 14.88) | 25.9 (-16.5, 31.5) |
|  |  | e2/e4 | 37 | 0.031 | 8.36 (1.42, 19.58) | 2.7 (1.0, 2.9) |
|  |  | e3/e4 | 591 | 0.488 | 12.39 (2.80, 21.89) | 44.9 (32.0, 46.6) |
|  |  | e4/e4 | 105 | 0.087 | 20.32 (8.67, 24.24) | 8.2 (7.7, 8.3) |
|  |  | total | 1203 |  |  | 85.1 (19.2, 93.9) |
| ADGC | Neuropath. confirmed AD, baseline prob 1% | e2/e2 | 5 | 0.001 | Ref. |  |
|  |  | e2/e3 | 113 | 0.028 | 2.94 (1.38, 5.71) | 1.9 (0.8, 2.3) |
|  |  | e3/e3 | 1273 | 0.317 | 7.21 (2.73, 16.81) | 27.3 (20.1, 29.8) |
|  |  | e2/e4 | 107 | 0.027 | 17.23 (4.42, 46.83) | 2.5 (2.1, 2.6) |
|  |  | e3/e4 | 1897 | 0.472 | 32.26 (12.48, 59.95) | 45.7 (43.4, 46.4) |
|  |  | e4/e4 | 623 | 0.155 | 70.81 (31.76, 92.23) | 15.3 (15.0, 15.3) |
|  |  | total | 4018 |  |  | 92.7 (81.4, 96.5) |
|  | Unconfirmed AD, baseline prob 1% | e2/e2 | 16 | 0.002 | Ref. |  |
|  |  | e2/e3 | 450 | 0.043 | 1.21 (0.83, 1.75) | 0.7 (-0.9, 1.9) |
|  |  | e3/e3 | 3604 | 0.346 | 1.91 (1.11, 3.26) | 16.4 (3.4, 23.9) |
|  |  | e2/e4 | 311 | 0.030 | 4.58 (2.22, 9.20) | 2.3 (1.6, 2.7) |
|  |  | e3/e4 | 4690 | 0.450 | 6.45 (3.44, 11.82) | 38.0 (31.9, 41.2) |
|  |  | e4/e4 | 1359 | 0.130 | 17.21 (9.29, 29.72) | 12.3 (11.6, 12.6) |
|  |  | total | 10430 |  |  | 69.8 (47.7, 82.2) |

| **Supplemental table 5: reorientating odds ratios to be with reference to e2/e2, rather than e3/e3, from ADGC data** | | | | | | |  |  |
| --- | --- | --- | --- | --- | --- | --- | --- | --- |
|  |  |  |  |  |  |  |  |  |
| **APOE genotype** | **Neuropathologically confirmed group** | | | | **Neuropathologically unconfirmed group** | | | |
|  | **Odds relative to e3/e3** | | **Odds relative to e2/e2** |  | **Odds relative to e3/e3** | | **Odds relative to e2/e2** | |
|  | **OR** | **95% CI** | **OR** | **95% CI** | **OR** | **95% CI** | **OR** | **95% CI** |
| e2/e2 | 0.13 | (0.05, 0.36) | Ref. | | 0.52 | (0.30, 0.90) | Ref. | |
| e2/e3 | 0.39 | (0.30, 0.50) | 3.00 | (1.39, 6.00) | 0.63 | (0.53, 0.75) | 1.21 | (0.83, 1.77) |
| e3/e3 | Ref. | | 7.69 | (2.78, 20.00) | Ref. | | 1.92 | (1.11, 3.33) |
| e2/e4 | 2.68 | (1.65, 4.36) | 20.62 | (4.58, 87.20) | 2.47 | (2.02, 3.01) | 4.75 | (2.24, 10.03) |
| e3/e4 | 6.13 | (5.08, 7.41) | 47.15 | (14.11, 148.00) | 3.55 | (3.17, 3.98) | 6.83 | (3.52, 13.27) |
| e4/e4 | 31.2 | (16.59, 58.75) | 240.15 | (46.08, 1175.00) | 10.70 | (9.12, 12.56) | 20.58 | (10.13, 41.87) |

| **Supplemental table 6. Partitioning of the PAF of AD attributable to *APOE* genotypes for the e3 and e4 alleles separately in the ADGC neuropathologically confirmed outcome dataset** | | | | |  |
| --- | --- | --- | --- | --- | --- |
| **Genotype** | | **Point estimate** | **lower CI** | **upper CI** |  |
| e3 | PAF for e2/e3, % | 1.9 | 0.78 | 2.32 |  |
|  | PAF for e3/e3, % | 27.3 | 20.07 | 29.80 |  |
|  | PAF for e3/e4, % | 45.75 | 43.43 | 46.43 |  |
|  | *ratio of e2/e4 to e3/e4 RRs ** | 5.86 | 33.85 | 0.77 |  |
|  | *Fraction of e3/e4 PAF attributable to e3* | 0.15 | 0.03 | 0.56 |  |
|  | share of e3/e4 PAF attributable to e3, % | 6.7 | 1.2 | 26.2 |  |
|  | Total PAF attributable to e3 (sum of PAFs for e2/e3, e3/e3 and share of e3/e4 PAF attributable to e3) | 35.8 | 22.1 | 58.3 |  |
| e4 | PAF for e2/e4, % | 2.5 | 2.1 | 2.6 |  |
|  | PAF for e4/e4, % | 15.3 | 15.0 | 15.3 |  |
|  | PAF for e3/e4, % | 45.7 | 43.4 | 46.4 |  |
|  | *ratio of e2/e4 to e3/e4 RRs *** | 5.86 | 0.77 | 33.85 |  |
|  | *Fraction of e3/e4 PAF attributable to e4* | 0.85 | 0.44 | 0.97 |  |
|  | share of e3/e4 PAF attributable to e4, % | 39.1 | 19.0 | 45.1 |  |
|  | Total PAF attributable to e4 (sum of PAFs for e2/e4, e4/e4 and share of e3/e4 PAF attributable to e4) | 56.9 | 36.0 | 63.0 |  |
|  | | | | | |
| *In the lower CI estimate for e3, the share of PAF for e3/e4 is calculated based on ratio of UCI for e2/e4 RR to LCI for e2/e3 RR; in the upper CI estimate for e3, the share of PAF for e3/e4 based on LCI for e2/e4 RR and UCI for e2/e3 | | | | | |
|  | | | | | |
| ** In the lower CI estimate for e4, the share of PAF for e3/e4 is calculated based on ratio of LCI for e2/e4 RR to UCI for e2/e3 RR; in the upper CI estimate for e4, the share of PAF for e3/e4 based on UCI for e2/e4 RR and LCI for e2/e3 | | | | | |

| **Supplemental table 7: AD PAF calculations from ADGC data assuming diffierent baseline probabilties of AD for OR-to-RR conversions** | | |
| --- | --- | --- |
|  |  |  |
| **Assumed baseline probability of AD in e2 homozygotes** | **PAF for AD estimated in neuropathologically confirmed sample** | **PAF for AD estimated in sample without neuropathological confirmation** |
| 1% | 92.7 (81.4, 96.5) | 69.8 (47.7, 82.2) |
| 2% | 91.8 (80.5, 95.5) | 69.1 (47.2, 81.4) |
| 3% | 90.8 (79.7, 94.5) | 68.4 (46.7, 80.5) |
| 5% | 88.9 (78.1, 92.6) | 66.9 (45.8, 78.9) |

**Supplemental table 8: PAF calculations for the top hits at 23 loci identified by a GWAS of Alzheimer's disease**


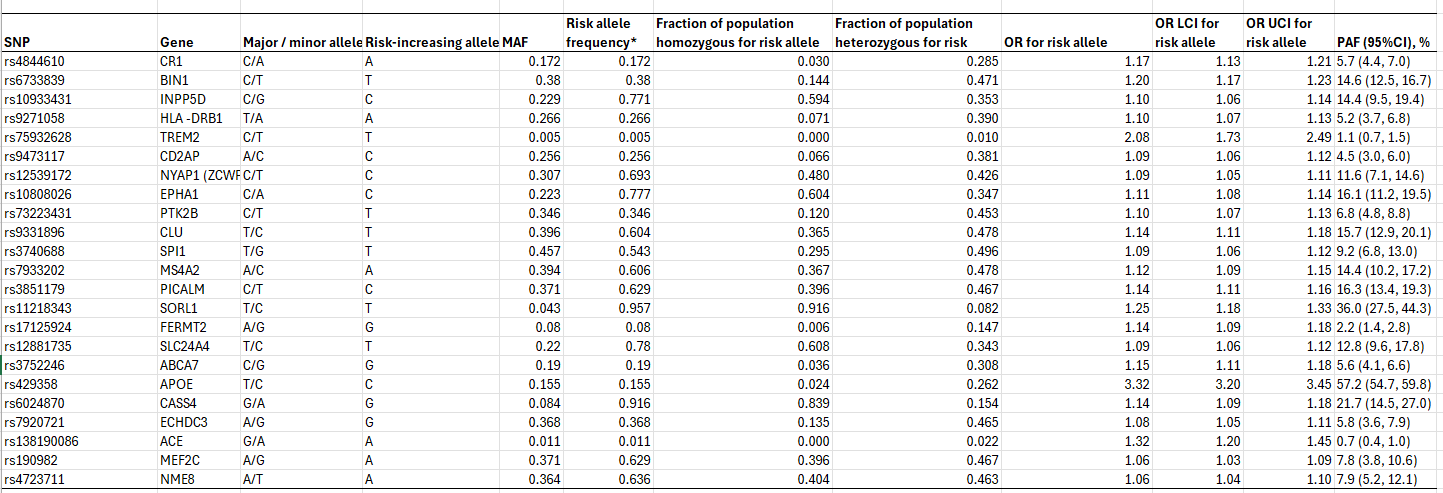


| Data adapted from Kunkle et al. *Nat Genet.* 2019 meta-analysis of stage 1 and 2 data (see table 1, https://doi.org/10.1038/s41588-019-0358-2) | | | | | | |
| --- | --- | --- | --- | --- | --- | --- |
|  |  |  |  |  |  |  |
| NB: the direction of associations needed reversing before PAF calculations for some of these SNPs where these were coded with the risk-decreasing alleles of genotypes as the effect allele in the original publication (and risk-increasing allele as the reference). In the original GWAS data, the minor alleles were coded as effect alleles in all instances | | | | | | |
|  |  |  |  |  |  |  |
| * AFs extracted from 1000 Genomes European sample (N=503), accessing its data on Ldlink via LDHap tool: <https://ldlink.nih.gov/?tab=ldhap> | | | | | | |

**Supplemental table 9: PAF calculations for the top hits at 65 loci identified by a GWAS of coronary artery disease**

| **Locus** | **Markername** | **Chr:Pos** | **Effect allele /** | **EAF in paper** | **EAF in 1KG Europeans *** | **Fraction of population homozygous for risk allele** | **Fraction of population heterozygous for risk allele** | **OR (95% CI)** | **PAF (95%CI), %** |
| --- | --- | --- | --- | --- | --- | --- | --- | --- | --- |
|  |  | **(hg19)** | **Other allele** |  |  |  |  |  |  |
| PCSK9 | rs11591147 | 1:55505647 | G/T | 0.984 | 0.979 | 0.959 | 0.041 | 1.25 (1.16,1.34) | 36.0 (25.5, 44.6) |
| PPAP2B | rs56170783 | 1:57016131 | A/C | 0.915 | 0.884 | 0.781 | 0.205 | 1.11 (1.08,1.14) | 17.6 (13.1, 21.8) |
| SORT1 | rs7528419 | 1:109817192 | A/G | 0.782 | 0.787 | 0.619 | 0.335 | 1.11 (1.09,1.14) | 16.1 (13.4, 20.1) |
| IL6R | rs6689306 | 1:154395946 | A/G | 0.435 | 0.442 | 0.196 | 0.493 | 1.05 (1.03,1.07) | 4.4 (2.6, 6.1) |
| MIA3 | rs67180937 | 1:222823743 | G/T | 0.684 | 0.738 | 0.544 | 0.387 | 1.07 (1.05,1.09) | 9.9 (7.2, 12.7) |
| AK097927 | rs16986953 | 2:19942473 | A/G | 0.073 | 0.088 | 0.008 | 0.161 | 1.11 (1.07,1.15) | 1.9 (1.2, 2.6) |
| APOB | rs585967 | 2:21270554 | C/A | 0.844 | 0.811 | 0.658 | 0.307 | 1.07 (1.04,1.09) | 10.8 (6.3, 13.7) |
| ABCG5/ ABCG8 | rs4299376 | 2:44072576 | G/T | 0.319 | 0.307 | 0.094 | 0.426 | 1.06 (1.04,1.08) | 3.6 (2.4, 4.8) |
| VAMP5/ VAMP8/ GGCX | rs7568458 | 2:85788175 | A/T | 0.452 | 0.478 | 0.228 | 0.499 | 1.06 (1.05,1.08) | 5.7 (4.7, 7.5) |
| ZEB2/ AC074093.1 | rs17678683 | 2:145286559 | G/T | 0.089 | 0.086 | 0.007 | 0.157 | 1.08 (1.05,1.11) | 1.4 (0.9, 1.9) |
| WDR12 | rs114123510 | 2:203831212 | A/T | 0.116 | 0.130 | 0.017 | 0.226 | 1.13 (1.10,1.15) | 3.3 (2.6, 3.8) |
| KCNJ13/ GIGYF2 | rs13003675 | 2:233584109 | T/C | 0.361 | 0.285 | 0.081 | 0.408 | 1.04 (1.03,1.06) | 2.3 (1.7, 3.4) |
| MRAS | rs139016349 | 3:138099161 | I/D | 0.163 | 0.153 | 0.023 | 0.259 | 1.08 (1.05,1.10) | 2.4 (1.5, 3.0) |
| REST/ NOA1 | rs72627509 | 4:57839051 | G/C | 0.199 | 0.183 | 0.033 | 0.299 | 1.06 (1.03,1.08) | 2.2 (1.1, 2.9) |
| EDNRA | rs6841581 | 4:148401190 | A/G | 0.153 | 0.146 | 0.021 | 0.249 | 1.07 (1.05,1.09) | 2.0 (1.4, 2.6) |
| GUCY1A3 | rs2306556 | 4:156638573 | A/G | 0.816 | 0.795 | 0.632 | 0.326 | 1.07 (1.04,1.09) | 10.6 (6.2, 13.5) |
| SLC22A4/ SLC22A5 | rs77335401 | 5:131759825 | C/T | 0.116 | 0.130 | 0.017 | 0.226 | 1.05 (1.03,1.08) | 1.3 (0.8, 2.1) |
| ADTRP/ C6orf105 | rs742115 | 6:11327021 | C/T | 0.484 | 0.485 | 0.235 | 0.500 | 1.04 (1.02,1.05) | 3.8 (1.9, 4.8) |
| PHACTR1 | rs9349379 | 6:12903957 | G/A | 0.407 | 0.401 | 0.161 | 0.480 | 1.11 (1.09,1.13) | 8.6 (7.1, 10.1) |
| C2 | rs3130683 | 6:31888367 | T/C | 0.860 | 0.893 | 0.797 | 0.191 | 1.08 (1.05,1.11) | 13.2 (8.5, 17.7) |
| ANKS1A | rs4472337 | 6:34769765 | T/C | 0.155 | 0.135 | 0.018 | 0.234 | 1.06 (1.03,1.08) | 1.6 (0.8, 2.1) |
| KCNK5 | rs56015508 | 6:39152041 | C/A | 0.794 | 0.771 | 0.594 | 0.353 | 1.06 (1.03,1.08) | 8.9 (4.5, 11.7) |
| TCF21 | rs12202017 | 6:134173151 | A/G | 0.702 | 0.707 | 0.500 | 0.414 | 1.07 (1.05,1.09) | 9.6 (6.9, 12.2) |
| SLC22A3/ LPAL2/ LPA/ PLG | rs10455872 | 6:161010118 | G/A | 0.065 | 0.074 | 0.005 | 0.137 | 1.31 (1.27,1.36) | 4.5 (3.9, 5.2) |
| HDAC9 | rs2107595 | 7:19049388 | A/G | 0.182 | 0.168 | 0.028 | 0.280 | 1.08 (1.06,1.10) | 2.7 (2.0, 3.3) |
| 7q22 | rs112370447 | 7:107176780 | T/C | 0.280 | 0.321 | 0.103 | 0.436 | 1.05 (1.03,1.07) | 3.2 (1.9, 4.4) |
| ZC3HC1 | rs11556924 | 7:129663496 | C/T | 0.657 | 0.623 | 0.388 | 0.470 | 1.07 (1.05,1.09) | 8.5 (6.1, 10.9) |
| NOS3 | rs3918226 | 7:150690176 | T/C | 0.071 | 0.098 | 0.010 | 0.177 | 1.13 (1.09,1.17) | 2.5 (1.7, 3.3) |
| LPL | rs2083636 | 8:19865263 | T/G | 0.742 | 0.715 | 0.511 | 0.408 | 1.05 (1.03,1.07) | 7.0 (4.2, 9.7) |
| TRIB1 | rs2954029 | 8:126490972 | A/T | 0.541 | 0.552 | 0.305 | 0.495 | 1.06 (1.04,1.08) | 6.5 (4.4, 8.6) |
| CDKN2BAS | rs2891168 | 9:22098619 | G/A | 0.487 | 0.492 | 0.242 | 0.500 | 1.19 (1.17,1.21) | 17.8 (16.0, 19.6) |
| SVEP1 | rs111245230 | 9:113169775 | C/T | 0.036 | 0.032 | 0.001 | 0.062 | 1.12 (1.07,1.17) | 0.8 (0.4, 1.1) |
| ABO | rs507666 | 9:136149399 | A/G | 0.192 | 0.186 | 0.035 | 0.303 | 1.08 (1.06,1.10) | 2.9 (2.2, 3.7) |
| KIAA1462 | rs1887318 | 10:30321598 | T/C | 0.428 | 0.434 | 0.188 | 0.491 | 1.06 (1.04,1.08) | 5.1 (3.4, 6.8) |
| CXCL12 | rs1870634 | 10:44480811 | G/T | 0.648 | 0.666 | 0.444 | 0.445 | 1.06 (1.05,1.08) | 7.8 (6.5, 10.3) |
| LIPA | rs2246942 | 10:91004886 | G/A | 0.349 | 0.361 | 0.130 | 0.461 | 1.08 (1.06,1.10) | 5.7 (4.3, 7.1) |
| CYP17A1/ CNNM2/ NT5C2 | rs11191416 | 10:104604916 | T/G | 0.890 | 0.908 | 0.824 | 0.167 | 1.08 (1.05,1.10) | 13.4 (8.6, 16.4) |
| SWAP70 | rs10840293 | 11:9751196 | A/G | 0.552 | 0.582 | 0.339 | 0.487 | 1.05 (1.03,1.07) | 5.7 (3.5, 8.0) |
| MRVI1/CTR9 | rs201267813 | 11:10284499 | I/D | 0.071 | 0.079 | 0.006 | 0.146 | 1.05 (1.02,1.08) | 0.8 (0.3, 1.3) |
| PDGFD | rs2839812 | 11:103673294 | T/A | 0.307 | 0.272 | 0.074 | 0.396 | 1.06 (1.04,1.08) | 3.2 (2.2, 4.3) |
| ZNF259/ APOA5/ APOA1 | rs964184 | 11:116648917 | G/C | 0.163 | 0.162 | 0.026 | 0.272 | 1.05 (1.03,1.08) | 1.6 (1.0, 2.6) |
| LRP1 | rs2229357 | 12:57843711 | G/A | 0.764 | 0.805 | 0.648 | 0.314 | 1.05 (1.03,1.07) | 7.8 (4.7, 10.7) |
| ATP2B1 | rs2681472 | 12:90008959 | G/A | 0.189 | 0.145 | 0.021 | 0.248 | 1.07 (1.05,1.09) | 2.0 (1.4, 2.6) |
| SH2B3 | rs10774625 | 12:111910219 | A/G | 0.491 | 0.477 | 0.228 | 0.499 | 1.07 (1.05,1.08) | 6.6 (4.7, 7.5) |
| KSR2 | rs11830157 | 12:118265441 | G/T | 0.382 | 0.408 | 0.166 | 0.483 | 1.03 (1.01,1.04) | 2.4 (0.8, 3.2) |
| SCARB1 | rs11057830 | 12:125307053 | A/G | 0.147 | 0.165 | 0.027 | 0.276 | 1.07 (1.05,1.10) | 2.3 (1.6, 3.3) |
| FLT1 | rs1924981 | 13:29022645 | T/C | 0.334 | 0.345 | 0.119 | 0.452 | 1.05 (1.03,1.06) | 3.4 (2.1, 4.1) |
| COL4A1/ COL4A2 | rs11617955 | 13:110818102 | T/A | 0.891 | 0.877 | 0.769 | 0.216 | 1.09 (1.06,1.12) | 14.5 (10.0, 18.9) |
| HHIPL1 | rs10139550 | 14:100145710 | G/C | 0.421 | 0.435 | 0.189 | 0.492 | 1.05 (1.04,1.07) | 4.3 (3.4, 6.0) |
| SMAD3 | rs72743461 | 15:67441750 | C/A | 0.783 | 0.785 | 0.616 | 0.338 | 1.07 (1.05,1.10) | 10.5 (7.6, 14.7) |
| ADAMTS7 | rs7164479 | 15:79123054 | T/C | 0.578 | 0.577 | 0.333 | 0.488 | 1.07 (1.06,1.09) | 7.9 (6.8, 10.1) |
| MFGE8/ ABHD2 | rs2083460 | 15:89574484 | T/C | 0.885 | 0.892 | 0.796 | 0.193 | 1.07 (1.05,1.10) | 11.7 (8.5, 16.2) |
| FURIN/ FES | rs2071382 | 15:91428197 | T/C | 0.464 | 0.471 | 0.222 | 0.498 | 1.06 (1.05,1.08) | 5.6 (4.7, 7.4) |
| CETP | rs247616 | 16:56989590 | C/T | 0.678 | 0.708 | 0.501 | 0.413 | 1.04 (1.03,1.06) | 5.6 (4.2, 8.3) |
| SMG6 | rs113348108 | 17:2088848 | D/I | 0.314 | **0.308** | 0.095 | 0.426 | 1.05 (1.03,1.07) | 3.1 (1.8, 4.3) |
| RAI1/ PEMT/ RASD1 | rs9897596 | 17:17593453 | T/C | 0.523 | 0.505 | 0.255 | 0.500 | 1.04 (1.02,1.06) | 4.0 (2.0, 6.0) |
| UBE2Z | rs4643373 | 17:47123423 | T/C | 0.724 | 0.743 | 0.552 | 0.382 | 1.05 (1.03,1.07) | 7.2 (4.4, 10.0) |
| BCAS3 | rs8068952 | 17:59286644 | G/C | 0.228 | 0.253 | 0.064 | 0.378 | 1.07 (1.05,1.10) | 3.5 (2.5, 5.0) |
| PMAIP1/ MC4R | rs35614134 | 18:57832856 | I/D | 0.248 | 0.242 | 0.058 | 0.367 | 1.04 (1.02,1.06) | 1.9 (1.0, 2.9) |
| ANGPTL4 | rs116843064 | 19:8429323 | G/A | 0.98 | 0.974 | 0.949 | 0.051 | 1.17 (1.10,1.25) | 26.8 (17.1, 36.0) |
| LDLR | rs6511720 | 19:11202306 | G/T | 0.884 | 0.890 | 0.792 | 0.196 | 1.14 (1.11,1.17) | 21.8 (17.6, 25.8) |
| ZNF507/ LOC400684 | rs10417115 | 19:33386556 | C/T | 0.061 | 0.057 | 0.003 | 0.108 | 1.07 (1.04,1.11) | 0.8 (0.5, 1.2) |
| APOE/APOC1 | rs7412 | 19:45412079 | C/T | 0.922 | 0.937 | 0.878 | 0.118 | 1.15 (1.12,1.19) | 23.8 (19.7, 29.0) |
| gene_desert/ KCNE2 | rs28451064 | 21:35593827 | A/G | 0.124 | 0.130 | 0.017 | 0.226 | 1.14 (1.11,1.17) | 3.6 (2.8, 4.3) |
| POM121L9P/ ADORA2A | rs180803 | 22:24658858 | G/T | 0.977 | 0.986 | 0.972 | 0.028 | 1.18 (1.12,1.24) | 28.1 (20.2, 35.0) |

* AFs extracted from 1000 Genomes European sample (N=503), accessing its data on Ldlink via LDHap tool: https://ldlink.nih.gov/?tab=ldhap. Some indel frequencies were not present in Ldlink, and these were extracted from gnomAD

**Supplemental table 10. FinnGen authorship**

| **Full Name** | **Affiliation** | **E-mail** | **Role 1** | **Role 2** |
| --- | --- | --- | --- | --- |
| Aarno Palotie | Institute for Molecular Medicine Finland (FIMM), HiLIFE, University of Helsinki, Helsinki, Finland; Broad Institute of MIT and Harvard; Massachusetts General Hospital, Boston, MA, United States | aarno.palotie@helsinki.fi | **Steering Committee** | **Steering Committee** |
| Mark Daly | Institute for Molecular Medicine Finland (FIMM), HiLIFE, University of Helsinki, Helsinki, Finland; Broad Institute of MIT and Harvard; Massachusetts General Hospital, Boston, MA, United States | mark.daly@helsinki.fi | **Steering Committee** | **Steering Committee** |
| Bridget Riley-Gills | Abbvie, Chicago, IL, United States | bridget.rileygillis@abbvie.com | **Steering Committee** | **Pharmaceutical companies** |
| Howard Jacob | Abbvie, Chicago, IL, United States | howard.jacob@abbvie.com | **Steering Committee** | **Pharmaceutical companies** |
| Coralie Viollet | Astra Zeneca, Cambridge, United Kingdom | coralie.viollet@astrazeneca.com | **Steering Committee** | **Pharmaceutical companies** |
| Slavé Petrovski | Astra Zeneca, Cambridge, United Kingdom | slav.petrovski@astrazeneca.com | **Steering Committee** | **Pharmaceutical companies** |
| Alix Berton | Bayer AG, Leverkusen, Germany | alix.berton@bayer.com | **Steering Committee** | **Pharmaceutical companies** |
| Santha Ramakrishnan | Bayer AG, Leverkusen, Germany | santha.ramakrishnan@bayer.com | **Steering Committee** | **Pharmaceutical companies** |
| Ellen Tsai | Biogen, Cambridge, MA, United States | ellen.tsai@biogen.com | **Steering Committee** | **Pharmaceutical companies** |
| Zhihao Ding | Boehringer Ingelheim, Ingelheim am Rhein, Germany | zhihao.ding@boehringer-ingelheim.com | **Steering Committee** | **Pharmaceutical companies** |
| Emily Holzinger | Bristol Myers Squibb, New York, NY, United States | emily.holzinger@bms.com | **Steering Committee** | **Pharmaceutical companies** |
| Robert Plenge | Bristol Myers Squibb, New York, NY, United States | robert.plenge@bms.com | **Steering Committee** | **Pharmaceutical companies** |
| Joseph Maranville | Bristol Myers Squibb, New York, NY, United States | joseph.maranville@bms.com | **Steering Committee** | **Pharmaceutical companies** |
| Mark McCarthy | Genentech, San Francisco, CA, United States | mccarthy.mark@gene.com | **Steering Committee** | **Pharmaceutical companies** |
| Rion Pendergrass | Genentech, San Francisco, CA, United States | penders2@gene.com | **Steering Committee** | **Pharmaceutical companies** |
| Jonathan Davitte | GlaxoSmithKline, Collegeville, PA, United States | jonathan.m.davitte@gsk.com | **Steering Committee** | **Pharmaceutical companies** |
| Simonne Longerich | Merck, Kenilworth, NJ, United States | simonne.longerich@merck.com | **Steering Committee** | **Pharmaceutical companies** |
| Anders Mälarstig | Pfizer, New York, NY, United States | anders.malarstig@pfizer.com | **Steering Committee** | **Pharmaceutical companies** |
| Anna Vlahiotis | Pfizer, New York, NY, United States | anna.vlahiotis@pfizer.com | **Steering Committee** | **Pharmaceutical companies** |
| Katherine Klinger | Translational Sciences, Sanofi R&D, Framingham, MA, USA | katherine.klinger@sanofi.com | **Steering Committee** | **Pharmaceutical companies** |
| Clement Chatelain | Translational Sciences, Sanofi R&D, Framingham, MA, USA | clement.chatelain@sanofi.com | **Steering Committee** | **Pharmaceutical companies** |
| Jorg Blankenstein | Translational Sciences, Sanofi R&D, Framingham, MA, USA | jorg.blankenstein@sanofi.com | **Steering Committee** | **Pharmaceutical companies** |
| Karol Estrada | Maze Therapeutics, San Francisco, CA, United States | kestrada@mazetx.com | **Steering Committee** | **Pharmaceutical companies** |
| Robert Graham | Maze Therapeutics, San Francisco, CA, United States | rgraham@mazetx.com | **Steering Committee** | **Pharmaceutical companies** |
| Dawn Waterworth | Johnson & Johnson Innovative Medicine, Spring House, PA, United States | dwaterwo@its.jnj.com | **Steering Committee** | **Pharmaceutical companies** |
| Chris O´Donnell | Novartis Institutes for BioMedical Research, Cambridge, MA, United States | chris.odonnell@novartis.com | **Steering Committee** | **Pharmaceutical companies** |
| Nicole Renaud | Novartis Institutes for BioMedical Research, Cambridge, MA, United States | nicole.renaud@novartis.com | **Steering Committee** | **Pharmaceutical companies** |
| Tomi P. Mäkelä | HiLIFE, University of Helsinki, Finland | tomi.makela@helsinki.fi | **Steering Committee** | **University of Helsinki & Biobanks** |
| Jaakko Kaprio | Institute for Molecular Medicine Finland (FIMM), HiLIFE, University of Helsinki, Helsinki, Finland | jaakko.kaprio@helsinki.fi | **Steering Committee** | **University of Helsinki & Biobanks** |
| Minna Ruddock | Arctic biobank / University of Oulu, Oulu, Finland | minna.ruddock@oulu.fi | **Steering Committee** | **University of Helsinki & Biobanks** |
| Petri Virolainen | Auria Biobank / University of Turku / Wellbeing Services County of Southwest Finland, Turku, Finland | petri.virolainen@tyks.fi | **Steering Committee** | **University of Helsinki & Biobanks** |
| Antti Hakanen | Auria Biobank / University of Turku / Wellbeing Services County of Southwest Finland, Turku, Finland | antti.hakanen@tyks.fi | **Steering Committee** | **University of Helsinki & Biobanks** |
| Terhi Kilpi | THL Biobank / Finnish Institute for Health and Welfare (THL), Helsinki, Finland | terhi.kilpi@thl.fi | **Steering Committee** | **University of Helsinki & Biobanks** |
| Markus Perola | THL Biobank / Finnish Institute for Health and Welfare (THL), Helsinki, Finland | markus.perola@thl.fi | **Steering Committee** | **University of Helsinki & Biobanks** |
| Jukka Partanen | Finnish Red Cross Blood Service / Finnish Hematology Registry and Clinical Biobank, Helsinki, Finland | jukka.partanen@veripalvelu.fi | **Steering Committee** | **University of Helsinki & Biobanks** |
| Taneli Raivio | Helsinki Biobank / Helsinki University and Hospital District of Helsinki and Uusimaa, Helsinki | taneli.raivio@hus.fi | **Steering Committee** | **University of Helsinki & Biobanks** |
| Raisa Serpi | Northern Finland Biobank Borealis / University of Oulu / Wellbeing services county of North Ostrobothnia, Oulu, Finland | raisa.serpi@pohde.fi | **Steering Committee** | **University of Helsinki & Biobanks** |
| Teija Kekonen | Northern Finland Biobank Borealis / University of Oulu / Wellbeing services county of North Ostrobothnia, Oulu, Finland | teija.kekonen@pohde.fi | **Steering Committee** | **University of Helsinki & Biobanks** |
| Kati Kristiansson | Finnish Clinical Biobank Tampere / University of Tampere / Wellbeing Services County of Pirkanmaa, Tampere, Finland | kati.kristiansson@pirha.fi | **Steering Committee** | **University of Helsinki & Biobanks** |
| Veli-Matti Kosma | Biobank of Eastern Finland / University of Eastern Finland / Wellbeing services county of North Savo, Kuopio, Finland | veli-matti.kosma@uef.fi | **Steering Committee** | **University of Helsinki & Biobanks** |
| Jari Laukkanen | Central Finland Biobank / University of Jyväskylä / Wellbeing Services County of Central Finland, Jyväskylä, Finland | jari.laukkanen@hyvaks.fi | **Steering Committee** | **University of Helsinki & Biobanks** |
| Tom Southerington | Finnish Biobank Cooperative - FINBB | tom.southerington@finbb.fi | **Steering Committee** | **University of Helsinki & Biobanks** |
| Outi Tuovila | Business Finland, Helsinki, Finland | outi.tuovila@businessfinland.fi | **Steering Committee** | **Other Experts/ Non-Voting Members** |
| Jeffrey Waring | Abbvie, Chicago, IL, United States | jeff.waring@abbvie.com | **Scientific Committee** | **Pharmaceutical companies** |
| Bridget Riley-Gillis | Abbvie, Chicago, IL, United States | bridget.rileygillis@abbvie.com | **Scientific Committee** | **Pharmaceutical companies** |
| Fedik Rahimov | Abbvie, Chicago, IL, United States | fedik.rahimov@abbvie.com | **Scientific Committee** | **Pharmaceutical companies** |
| Ioanna Tachmazidou | Astra Zeneca, Cambridge, United Kingdom | ioanna.tachmazidou@astrazeneca.com | **Scientific Committee** | **Pharmaceutical companies** |
| Alix Berton | Bayer AG, Leverkusen, Germany | alix.berton@bayer.com | **Scientific Committee** | **Pharmaceutical companies** |
| Santha Ramakrishnan | Bayer AG, Leverkusen, Germany | santha.ramakrishnan@bayer.com | **Scientific Committee** | **Pharmaceutical companies** |
| Ellen Tsai | Biogen, Cambridge, MA, United States | ellen.tsai@biogen.com | **Scientific Committee** | **Pharmaceutical companies** |
| Zhihao Ding | Boehringer Ingelheim, Ingelheim am Rhein, Germany | zhihao.ding@boehringer-ingelheim.com | **Scientific Committee** | **Pharmaceutical companies** |
| Marc Jung | Boehringer Ingelheim, Ingelheim am Rhein, Germany | marc_oliver.jung@boehringer-ingelheim.com | **Scientific Committee** | **Pharmaceutical companies** |
| Hanati Tuoken | Boehringer Ingelheim, Ingelheim am Rhein, Germany | hanati.tuoken@boehringer-ingelheim.com | **Scientific Committee** | **Pharmaceutical companies** |
| Shameek Biswas | Bristol Myers Squibb, New York, NY, United States | Shameek.Biswas@bms.com | **Scientific Committee** | **Pharmaceutical companies** |
| Benjamin Sun | Bristol Myers Squibb, New York, NY, United States | Benjamin.Sun@bms.com | **Scientific Committee** | **Pharmaceutical companies** |
| Rion Pendergrass | Genentech, San Francisco, CA, United States | penders2@gene.com | **Scientific Committee** | **Pharmaceutical companies** |
| Jonathan Davitte | GlaxoSmithKline, Collegeville, PA, United States | jonathan.m.davitte@gsk.com | **Scientific Committee** | **Pharmaceutical companies** |
| Neha Raghavan | Merck, Kenilworth, NJ, United States | neha.raghavan@merck.com | **Scientific Committee** | **Pharmaceutical companies** |
| Adriana Huertas-Vazquez | Merck, Kenilworth, NJ, United States | adriana.huertas.vazquez@merck.com | **Scientific Committee** | **Pharmaceutical companies** |
| Jae-Hoon Sul | Merck, Kenilworth, NJ, United States | jae.hoon.sul@merck.com | **Scientific Committee** | **Pharmaceutical companies** |
| Anders Mälarstig | Pfizer, New York, NY, United States | anders.malarstig@pfizer.com | **Scientific Committee** | **Pharmaceutical companies** |
| Xinli Hu | Pfizer, New York, NY, United States | xinli.hu@pfizer.com | **Scientific Committee** | **Pharmaceutical companies** |
| Åsa Hedman | Pfizer, New York, NY, United States | asa.hedman@pfizer.com | **Scientific Committee** | **Pharmaceutical companies** |
| Katherine Klinger | Translational Sciences, Sanofi R&D, Framingham, MA, USA | katherine.klinger@sanofi.com | **Scientific Committee** | **Pharmaceutical companies** |
| Robert Graham | Maze Therapeutics, San Francisco, CA, United States | rgraham@mazetx.com | **Scientific Committee** | **Pharmaceutical companies** |
| Dawn Waterworth | Johnson & Johnson Innovative Medicine, Spring House, PA, United States | dwaterwo@its.jnj.com | **Scientific Committee** | **Pharmaceutical companies** |
| Nicole Renaud | Novartis Institutes for BioMedical Research, Cambridge, MA, United States | nicole.renaud@novartis.com | **Scientific Committee** | **Pharmaceutical companies** |
| Ma´en Obeidat | Novartis Institutes for BioMedical Research, Cambridge, MA, United States | maen.obeidat@novartis.com | **Scientific Committee** | **Pharmaceutical companies** |
| Jonathan Chung | Novartis Institutes for BioMedical Research, Cambridge, MA, United States | jonathan.chung@novartis.com | **Scientific Committee** | **Pharmaceutical companies** |
| Jonas Zierer | Novartis Institutes for BioMedical Research, Cambridge, MA, United States | jonas.zierer@novartis.com | **Scientific Committee** | **Pharmaceutical companies** |
| Mari Niemi | Novartis Institutes for BioMedical Research, Cambridge, MA, United States | mari.niemi@novartis.com | **Scientific Committee** | **Pharmaceutical companies** |
| Samuli Ripatti | Institute for Molecular Medicine Finland (FIMM), HiLIFE, University of Helsinki, Helsinki, Finland | samuli.ripatti@helsinki.fi | **Scientific Committee** | **University of Helsinki & Biobanks** |
| Johanna Schleutker | Auria Biobank / University of Turku / Wellbeing Services County of Southwest Finland, Turku, Finland | johanna.schleutker@utu.fi | **Scientific Committee** | **University of Helsinki & Biobanks** |
| Markus Perola | THL Biobank / Finnish Institute for Health and Welfare (THL), Helsinki, Finland | markus.perola@thl.fi | **Scientific Committee** | **University of Helsinki & Biobanks** |
| Mikko Arvas | Finnish Red Cross Blood Service / Finnish Hematology Registry and Clinical Biobank, Helsinki, Finland | mikko.arvas@veripalvelu.fi | **Scientific Committee** | **University of Helsinki & Biobanks** |
| Olli Carpén | Helsinki Biobank / Helsinki University and Hospital District of Helsinki and Uusimaa, Helsinki | olli.carpen@helsinki.fi | **Scientific Committee** | **University of Helsinki & Biobanks** |
| Reetta Hinttala | Northern Finland Biobank Borealis / University of Oulu / Wellbeing services county of North Ostrobothnia, Oulu, Finland | reetta.hinttala@oulu.fi | **Scientific Committee** | **University of Helsinki & Biobanks** |
| Johannes Kettunen | Northern Finland Biobank Borealis / University of Oulu / Wellbeing services county of North Ostrobothnia, Oulu, Finland | johannes.kettunen@oulu.fi | **Scientific Committee** | **University of Helsinki & Biobanks** |
| Arto Mannermaa | Biobank of Eastern Finland / University of Eastern Finland / Wellbeing services county of North Savo, Kuopio, Finland | arto.mannermaa@uef.fi | **Scientific Committee** | **University of Helsinki & Biobanks** |
| Katriina Aalto-Setälä | Faculty of Medicine and Health Technology, Tampere University, Tampere, Finland | katriina.aalto-setala@tuni.fi | **Scientific Committee** | **University of Helsinki & Biobanks** |
| Mika Kähönen | Finnish Clinical Biobank Tampere / University of Tampere / Wellbeing Services County of Pirkanmaa, Tampere, Finland | mika.kahonen@uta.fi | **Scientific Committee** | **University of Helsinki & Biobanks** |
| Jari Laukkanen | Central Finland Biobank / University of Jyväskylä / Wellbeing Services County of Central Finland, Jyväskylä, Finland | jari.laukkanen@hyvaks.fi | **Scientific Committee** | **University of Helsinki & Biobanks** |
| Johanna Mäkelä | FINBB - Finnish biobank cooperative | johanna.makela@finbb.fi | **Scientific Committee** | **University of Helsinki & Biobanks** |
| Lila Kallio | Auria Biobank / University of Turku / Wellbeing Services County of Southwest Finland, Turku, Finland | Lila.Kallio@tyks.fi | **Biobank directors** | **Biobank directors** |
| Tiina Wahlfors | THL Biobank / Finnish Institute for Health and Welfare (THL), Helsinki, Finland | tiina.wahlfors@thl.fi | **Biobank directors** | **Biobank directors** |
| Jukka Partanen | Finnish Red Cross Blood Service / Finnish Hematology Registry and Clinical Biobank, Helsinki, Finland | jukka.partanen@veripalvelu.fi | **Biobank directors** | **Biobank directors** |
| Eero Punkka | Helsinki Biobank / Helsinki University and Hospital District of Helsinki and Uusimaa, Helsinki | eero.punkka@hus.fi | **Biobank directors** | **Biobank directors** |
| Raisa Serpi | Northern Finland Biobank Borealis / University of Oulu / Wellbeing services county of North Ostrobothnia, Oulu, Finland | raisa.serpi@pohde.fi | **Biobank directors** | **Biobank directors** |
| Sanna Siltanen | Finnish Clinical Biobank Tampere / University of Tampere / Wellbeing Services County of Pirkanmaa, Tampere, Finland | sanna.siltanen@pirha.fi | **Biobank directors** | **Biobank directors** |
| Veli-Matti Kosma | Biobank of Eastern Finland / University of Eastern Finland / Wellbeing services county of North Savo, Kuopio, Finland | veli-matti.kosma@uef.fi | **Biobank directors** | **Biobank directors** |
| Tiina Jokela | Central Finland Biobank / University of Jyväskylä / Wellbeing Services County of Central Finland, Jyväskylä, Finland | tiina.a.jokela@jyu.fi | **Biobank directors** | **Biobank directors** |
| Anu Jalanko | Institute for Molecular Medicine Finland (FIMM), HiLIFE, University of Helsinki, Helsinki, Finland | anu.jalanko@helsinki.fi | **FinnGen Teams** | **Administration** |
| Auli Toivola | Institute for Molecular Medicine Finland (FIMM), HiLIFE, University of Helsinki, Helsinki, Finland | auli.toivola@helsinki.fi | **FinnGen Teams** | **Administration** |
| Denise Öller | Institute for Molecular Medicine Finland (FIMM), HiLIFE, University of Helsinki, Helsinki, Finland | denise.oller@helsinki.fi | **FinnGen Teams** | **Administration** |
| Helen Cooper | Institute for Molecular Medicine Finland (FIMM), HiLIFE, University of Helsinki, Helsinki, Finland | helen.cooper@helsinki.fi | **FinnGen Teams** | **Administration** |
| Mervi Aavikko | Institute for Molecular Medicine Finland (FIMM), HiLIFE, University of Helsinki, Helsinki, Finland | mervi.aavikko@helsinki.fi | **FinnGen Teams** | **Administration** |
| Risto Kajanne | Institute for Molecular Medicine Finland (FIMM), HiLIFE, University of Helsinki, Helsinki, Finland | risto.kajanne@helsinki.fi | **FinnGen Teams** | **Administration** |
| Rodos Rodosthenous | Institute for Molecular Medicine Finland (FIMM), HiLIFE, University of Helsinki, Helsinki, Finland | rodos.rodosthenous@helsinki.fi | **FinnGen Teams** | **Administration** |
| Sofia Kuitunen | University of Helsinki, Helsinki, Finland | sofia.kuitunen@helsinki.fi | **FinnGen Teams** | **Administration** |
| Tarja Laitinen | Institute for Molecular Medicine Finland (FIMM), HiLIFE, University of Helsinki, Helsinki, Finland | tarja.laitinen@helsinki.fi | **FinnGen Teams** | **Administration** |
| Arto Lehisto | Institute for Molecular Medicine Finland (FIMM), HiLIFE, University of Helsinki, Helsinki, Finland | arto.lehisto@helsinki.fi | **FinnGen Teams** | **Analysis** |
| Hafiz Sikandar | Institute for Molecular Medicine Finland (FIMM), HiLIFE, University of Helsinki, Helsinki, Finland | hafiz.sikandar@helsinki.fi | **FinnGen Teams** | **Analysis** |
| Juha Karjalainen | Institute for Molecular Medicine Finland (FIMM), HiLIFE, University of Helsinki, Helsinki, Finland | juha.karjalainen@helsinki.fi | **FinnGen Teams** | **Analysis** |
| Juha Mehtonen | Institute for Molecular Medicine Finland (FIMM), HiLIFE, University of Helsinki, Helsinki, Finland | juha.mehtonen@helsinki.fi | **FinnGen Teams** | **Analysis** |
| Masahiro Kanai | Broad Institute, Cambridge, MA, United States | mkanai@broadinstitute.org | **FinnGen Teams** | **Analysis** |
| Mitja Kurki | Institute for Molecular Medicine Finland (FIMM), HiLIFE, University of Helsinki, Helsinki, Finland; Broad Institute, Cambridge, MA, United States | mkurki@broadinstitute.org | **FinnGen Teams** | **Analysis** |
| Mutaamba Maasha | Broad Institute, Cambridge, MA, United States | mmaasha@broadinstitute.org | **FinnGen Teams** | **Analysis** |
| Pietro Della Briotta Parolo | Institute for Molecular Medicine Finland (FIMM), HiLIFE, University of Helsinki, Helsinki, Finland | pietro.dellabriottaparolo@helsinki.fi | **FinnGen Teams** | **Analysis** |
| Samuel Jones | Institute for Molecular Medicine Finland (FIMM), HiLIFE, University of Helsinki, Helsinki, Finland | samuel.jones@helsinki.fi | **FinnGen Teams** | **Analysis** |
| Sanni Ruotsalainen | Institute for Molecular Medicine Finland (FIMM), HiLIFE, University of Helsinki, Helsinki, Finland | sanni.ruotsalainen@helsinki.fi | **FinnGen Teams** | **Analysis** |
| Susanna Lemmelä | Institute for Molecular Medicine Finland (FIMM), HiLIFE, University of Helsinki, Helsinki, Finland | susanna.lemmela@helsinki.fi | **FinnGen Teams** | **Analysis** |
| Wei Zhou | Broad Institute, Cambridge, MA, United States | wzhou@broadinstitute.org | **FinnGen Teams** | **Analysis** |
| Aki Havulinna | Finnish Institute for Health and Welfare (THL), Helsinki, Finland | aki.havulinna@thl.fi | **FinnGen Teams** | **Clinical Endpoint Development** |
| L. Elisa Lahtela | Institute for Molecular Medicine Finland (FIMM), HiLIFE, University of Helsinki, Helsinki, Finland | laura.lahtela@helsinki.fi | **FinnGen Teams** | **Clinical Endpoint Development** |
| Mari Kaunisto | Institute for Molecular Medicine Finland (FIMM), HiLIFE, University of Helsinki, Helsinki, Finland | mari.kaunisto@helsinki.fi | **FinnGen Teams** | **Communication** |
| Awaisa Ghazal | Institute for Molecular Medicine Finland (FIMM), HiLIFE, University of Helsinki, Helsinki, Finland | awaisa.ghazal@helsinki.fi | **FinnGen Teams** | **E-Science** |
| Elina Kilpeläinen | Institute for Molecular Medicine Finland (FIMM), HiLIFE, University of Helsinki, Helsinki, Finland | elina.kilpelainen@helsinki.fi | **FinnGen Teams** | **E-Science** |
| Jaska Uimonen | Institute for Molecular Medicine Finland (FIMM), HiLIFE, University of Helsinki, Helsinki, Finland | jaska.uimonen@helsinki.fi | **FinnGen Teams** | **E-Science** |
| Oluwaseun Alexander Dada | Institute for Molecular Medicine Finland (FIMM), HiLIFE, University of Helsinki, Helsinki, Finland | alexander.dada@helsinki.fi | **FinnGen Teams** | **E-Science** |
| Rigbe Weldatsadik | Institute for Molecular Medicine Finland (FIMM), HiLIFE, University of Helsinki, Helsinki, Finland | rigbe.weldatsadik@helsinki.fi | **FinnGen Teams** | **E-Science** |
| Sanni Ruotsalainen | Institute for Molecular Medicine Finland (FIMM), HiLIFE, University of Helsinki, Helsinki, Finland | sanni.ruotsalainen@helsinki.fi | **FinnGen Teams** | **E-Science** |
| Tianduanyi Wang | Institute for Molecular Medicine Finland (FIMM), HiLIFE, University of Helsinki, Helsinki, Finland | tianduanyi.wang@helsinki.fi | **FinnGen Teams** | **E-Science** |
| Timo P. Sipilä | Institute for Molecular Medicine Finland (FIMM), HiLIFE, University of Helsinki, Helsinki, Finland | timo.p.sipila@helsinki.fi | **FinnGen Teams** | **E-Science** |
| Kati Donner | Institute for Molecular Medicine Finland (FIMM), HiLIFE, University of Helsinki, Helsinki, Finland | kati.donner@helsinki.fi | **FinnGen Teams** | **Genotyping** |
| Anu Loukola | Helsinki Biobank / Helsinki University and Hospital District of Helsinki and Uusimaa, Helsinki | anu.loukola@hus.fi | **FinnGen Teams** | **Sample Collection Coordination** |
| Päivi Ingalsuo | THL Biobank / Finnish Institute for Health and Welfare (THL), Helsinki, Finland | paivi.ingalsuo@thl.fi | **FinnGen Teams** | **Sample Logistics** |
| Arto Pietilä | THL Biobank / Finnish Institute for Health and Welfare (THL), Helsinki, Finland | arto.pietila@thl.fi | **FinnGen Teams** | **Registry Data Operations** |
| Sami Koskelainen | THL Biobank / Finnish Institute for Health and Welfare (THL), Helsinki, Finland | sami.koskelainen@thl.fi | **FinnGen Teams** | **Registry Data Operations** |
| Susanna Lemmelä | Institute for Molecular Medicine Finland (FIMM), HiLIFE, University of Helsinki, Helsinki, Finland | susanna.lemmela@helsinki.fi | **FinnGen Teams** | **Registry Data Operations** |
| Teemu Paajanen | THL Biobank / Finnish Institute for Health and Welfare (THL), Helsinki, Finland | teemu.paajanen@thl.fi | **FinnGen Teams** | **Registry Data Operations** |
| Tero Hiekkalinna | THL Biobank / Finnish Institute for Health and Welfare (THL), Helsinki, Finland | tero.hiekkalinna@helsinki.fi | **FinnGen Teams** | **Registry Data Operations** |
| Priit Palta | Institute for Molecular Medicine Finland (FIMM), HiLIFE, University of Helsinki, Helsinki, Finland | priit.palta@helsinki.fi | **FinnGen Teams** | **Sequencing Informatics** |
| Dawit A. Yohannes | Institute for Molecular Medicine Finland (FIMM), HiLIFE, University of Helsinki, Helsinki, Finland | dawit.yohannes@helsinki.fi | **FinnGen Teams** | **Phenotype team** |
| Harri Siirtola | University of Tampere, Tampere, Finland | harri.siirtola@tuni.fi | **FinnGen Teams** | **Phenotype team** |
| Javier Gracia-Tabuenca | University of Tampere, Tampere, Finland | javier.graciatabuenca@tuni.fi | **FinnGen Teams** | **Phenotype team** |
| Marika Kaakinen | Institute for Molecular Medicine Finland (FIMM), HiLIFE, University of Helsinki, Helsinki, Finland | marika.kaakinen@helsinki.fi | **FinnGen Teams** | **Phenotype team** |
| Mary Pat Reeve | Institute for Molecular Medicine Finland (FIMM), HiLIFE, University of Helsinki, Helsinki, Finland | mary.reeve@helsinki.fi | **FinnGen Teams** | **Phenotype team** |
| Shanmukha Sampath Padmanabhuni | Institute for Molecular Medicine Finland (FIMM), HiLIFE, University of Helsinki, Helsinki, Finland | sam.padmanabhuni@helsinki.fi | **FinnGen Teams** | **Phenotype team** |
| Shuang Luo | Institute for Molecular Medicine Finland (FIMM), HiLIFE, University of Helsinki, Helsinki, Finland | shuang.luo@helsinki.fi | **FinnGen Teams** | **Phenotype team** |
| Vincent Llorens | Institute for Molecular Medicine Finland (FIMM), HiLIFE, University of Helsinki, Helsinki, Finland | vincent.llorens@helsinki.fi | **FinnGen Teams** | **Phenotype team** |
| Iina Laak | Institute for Molecular Medicine Finland (FIMM), HiLIFE, University of Helsinki, Helsinki, Finland | iina.laak@helsinki.fi | **FinnGen Teams** | **Data protection officer** |
| Jaakko Tyrmi | University of Oulu, Oulu, Finland / University of Tampere, Tampere, Finland | jaakko.tyrmi@oulu.fi | **FinnGen Teams** | **FinnGen local support person** |
| Janne Isojärvi | University of Turku, Turku, Finland | jaheis@utu.fi | **FinnGen Teams** | **FinnGen local support person** |
| Tero Sievänen | University of Eastern Finland, Kuopio, Finland | tero.sievanen@uef.fi | **FinnGen Teams** | **FinnGen local support person** |
| Timo Pohjonen | University of Jyväskylä, Jyväskylä, Finland | timo.pohjonen@hyvaks.fi | **FinnGen Teams** | **FinnGen local support person** |
| Vidal Fey | University of Tampere, Tampere, Finland | vidal.fey@tuni.fi | **FinnGen Teams** | **FinnGen local support person** |
| Johanna Mäkelä | Finnish Biobank Cooperative - FINBB | johanna.makela@finbb.fi | **FinnGen Teams** | **FINBB - Finnish biobank cooperative** |
| Pauli Wihuri | Finnish Biobank Cooperative - FINBB | pauli.wihuri@finbb.fi | **FinnGen Teams** | **FINBB - Finnish biobank cooperative** |
| Tom Southerington | Finnish Biobank Cooperative - FINBB | tom.southerington@finbb.fi | **FinnGen Teams** | **FINBB - Finnish biobank cooperative** |
| Meri Lähteenmäki | Finnish Biobank Cooperative - FINBB | meri.lahteenmaki@finbb.fi | **FinnGen Teams** | **FINBB - Finnish biobank cooperative** |
| Reetta Kälviäinen | Wellbeing services county of North Savo, Kuopio, Finland | reetta.kalviainen@kuh.fi | **Clinical Groups (FinnGen phases 1&2)** | **Neurology Group** |
| Valtteri Julkunen | Wellbeing services county of North Savo, Kuopio, Finland | valtteri.julkunen@kuh.fi | **Clinical Groups (FinnGen phases 1&2)** | **Neurology Group** |
| Hilkka Soininen | Wellbeing services county of North Savo, Kuopio, Finland | hilkka.soininen@uef.fi | **Clinical Groups (FinnGen phases 1&2)** | **Neurology Group** |
| Anne Remes | Wellbeing services county of North Ostrobothnia, Oulu, Finland | anne.remes@oulu.fi | **Clinical Groups (FinnGen phases 1&2)** | **Neurology Group** |
| Mikko Hiltunen | University of Eastern Finland, Kuopio, Finland | mikko.hiltunen@uef.fi | **Clinical Groups (FinnGen phases 1&2)** | **Neurology Group** |
| Jukka Peltola | Wellbeing Services County of Pirkanmaa, Tampere, Finland | jukka.peltola@pirha.fi | **Clinical Groups (FinnGen phases 1&2)** | **Neurology Group** |
| Minna Raivio | Hospital District of Helsinki and Uusimaa, Helsinki, Finland | minna.raivio@geri.fi | **Clinical Groups (FinnGen phases 1&2)** | **Neurology Group** |
| Pentti Tienari | Hospital District of Helsinki and Uusimaa, Helsinki, Finland | pentti.tienari@hus.fi | **Clinical Groups (FinnGen phases 1&2)** | **Neurology Group** |
| Juha Rinne | Wellbeing Services County of Southwest Finland, Turku, Finland | juha.rinne@tyks.fi | **Clinical Groups (FinnGen phases 1&2)** | **Neurology Group** |
| Roosa Kallionpää | Wellbeing Services County of Southwest Finland, Turku, Finland | roosa.kallionpaa@tyks.fi | **Clinical Groups (FinnGen phases 1&2)** | **Neurology Group** |
| Juulia Partanen | Institute for Molecular Medicine Finland, HiLIFE, University of Helsinki, Finland | juulia.partanen@helsinki.fi | **Clinical Groups (FinnGen phases 1&2)** | **Neurology Group** |
| Adam Ziemann | Abbvie, Chicago, IL, United States | adam.ziemann@abbvie.com | **Clinical Groups (FinnGen phases 1&2)** | **Neurology Group** |
| Nizar Smaoui | Abbvie, Chicago, IL, United States | nizar.smaoui@abbvie.com | **Clinical Groups (FinnGen phases 1&2)** | **Neurology Group** |
| Anne Lehtonen | Abbvie, Chicago, IL, United States | anne.lehtonen@abbvie.com | **Clinical Groups (FinnGen phases 1&2)** | **Neurology Group** |
| Susan Eaton | Biogen, Cambridge, MA, United States | susan.eaton@biogen.com | **Clinical Groups (FinnGen phases 1&2)** | **Neurology Group** |
| Shameek Biswas | Bristol Myers Squibb, New York, NY, United States | shameek.biswas@bms.com | **Clinical Groups (FinnGen phases 1&2)** | **Neurology Group** |
| Natalie Bowers | Genentech, San Francisco, CA, United States | bowersn1@gene.com | **Clinical Groups (FinnGen phases 1&2)** | **Neurology Group** |
| Edmond Teng | Genentech, San Francisco, CA, United States | teng.edmond@gene.com | **Clinical Groups (FinnGen phases 1&2)** | **Neurology Group** |
| Rion Pendergrass | Genentech, San Francisco, CA, United States | penders2@gene.com | **Clinical Groups (FinnGen phases 1&2)** | **Neurology Group** |
| Fanli Xu | GlaxoSmithKline, Brentford, United Kingdom | chun-fang.2.xu@gsk.com | **Clinical Groups (FinnGen phases 1&2)** | **Neurology Group** |
| Laura Addis | GlaxoSmithKline, Brentford, United Kingdom | laura.x.addis@gsk.com | **Clinical Groups (FinnGen phases 1&2)** | **Neurology Group** |
| John Eicher | GlaxoSmithKline, Brentford, United Kingdom | john.d.eicher@gsk.com | **Clinical Groups (FinnGen phases 1&2)** | **Neurology Group** |
| Qingqin S Li | Johnson & Johnson Innovative Medicine, Titusville, NJ 08560, United States | QLi2@its.jnj.com | **Clinical Groups (FinnGen phases 1&2)** | **Neurology Group** |
| Karen He | Johnson & Johnson Innovative Medicine, Spring House, PA, United States | khe2@its.jnj.com | **Clinical Groups (FinnGen phases 1&2)** | **Neurology Group** |
| Ekaterina Khramtsova | Johnson & Johnson Innovative Medicine, Spring House, PA, United States | ekhramts@its.jnj.com | **Clinical Groups (FinnGen phases 1&2)** | **Neurology Group** |
| Neha Raghavan | Merck, Kenilworth, NJ, United States | neha.raghavan@merck.com | **Clinical Groups (FinnGen phases 1&2)** | **Neurology Group** |
| Martti Färkkilä | Hospital District of Helsinki and Uusimaa, Helsinki, Finland | martti.farkkila@hus.fi | **Clinical Groups (FinnGen phases 1&2)** | **Gastroenterology Group** |
| Jukka Koskela | Hospital District of Helsinki and Uusimaa, Helsinki, Finland | jukka.koskela@helsinki.fi | **Clinical Groups (FinnGen phases 1&2)** | **Gastroenterology Group** |
| Sampsa Pikkarainen | Hospital District of Helsinki and Uusimaa, Helsinki, Finland | sampsa.pikkarainen@hus.fi | **Clinical Groups (FinnGen phases 1&2)** | **Gastroenterology Group** |
| Airi Jussila | Wellbeing Services County of Pirkanmaa, Tampere, Finland | airi.jussila@pirha.fi | **Clinical Groups (FinnGen phases 1&2)** | **Gastroenterology Group** |
| Katri Kaukinen | Wellbeing Services County of Pirkanmaa, Tampere, Finland | katri.kaukinen@tuni.fi | **Clinical Groups (FinnGen phases 1&2)** | **Gastroenterology Group** |
| Timo Blomster | Wellbeing services county of North Ostrobothnia, Oulu, Finland | timo.blomster@pohde.fi | **Clinical Groups (FinnGen phases 1&2)** | **Gastroenterology Group** |
| Mikko Kiviniemi | Wellbeing services county of North Savo, Kuopio, Finland | mikko.kiviniemi@kuh.fi | **Clinical Groups (FinnGen phases 1&2)** | **Gastroenterology Group** |
| Markku Voutilainen | Wellbeing Services County of Southwest Finland, Turku, Finland | markku.voutilainen@tyks.fi | **Clinical Groups (FinnGen phases 1&2)** | **Gastroenterology Group** |
| Mark Daly | Institute for Molecular Medicine Finland (FIMM), HiLIFE, University of Helsinki, Helsinki, Finland; Broad Institute of MIT and Harvard; Massachusetts General Hospital, Boston, MA, United States | mark.daly@helsinki.fi | **Clinical Groups (FinnGen phases 1&2)** | **Gastroenterology Group** |
| Jeffrey Waring | Abbvie, Chicago, IL, United States | jeff.waring@abbvie.com | **Clinical Groups (FinnGen phases 1&2)** | **Gastroenterology Group** |
| Nizar Smaoui | Abbvie, Chicago, IL, United States | nizar.smaoui@abbvie.com | **Clinical Groups (FinnGen phases 1&2)** | **Gastroenterology Group** |
| Fedik Rahimov | Abbvie, Chicago, IL, United States | fedik.rahimov@abbvie.com | **Clinical Groups (FinnGen phases 1&2)** | **Gastroenterology Group** |
| Anne Lehtonen | Abbvie, Chicago, IL, United States | anne.lehtonen@abbvie.com | **Clinical Groups (FinnGen phases 1&2)** | **Gastroenterology Group** |
| Tim Lu | Genentech, San Francisco, CA, United States | lut8@gene.com | **Clinical Groups (FinnGen phases 1&2)** | **Gastroenterology Group** |
| Natalie Bowers | Genentech, San Francisco, CA, United States | bowersn1@gene.com | **Clinical Groups (FinnGen phases 1&2)** | **Gastroenterology Group** |
| Rion Pendergrass | Genentech, San Francisco, CA, United States | penders2@gene.com | **Clinical Groups (FinnGen phases 1&2)** | **Gastroenterology Group** |
| Linda McCarthy | GlaxoSmithKline, Brentford, United Kingdom | linda.c.mccarthy@gsk.com | **Clinical Groups (FinnGen phases 1&2)** | **Gastroenterology Group** |
| Amy Hart | Johnson & Johnson Innovative Medicine, Spring House, PA, United States | ahart13@its.jnj.com | **Clinical Groups (FinnGen phases 1&2)** | **Gastroenterology Group** |
| Meijian Guan | Johnson & Johnson Innovative Medicine, Spring House, PA, United States | mguan4@its.jnj.com | **Clinical Groups (FinnGen phases 1&2)** | **Gastroenterology Group** |
| Jason Miller | Merck, Kenilworth, NJ, United States | jason.miller4@merck.com | **Clinical Groups (FinnGen phases 1&2)** | **Gastroenterology Group** |
| Kirsi Kalpala | Pfizer, New York, NY, United States | kirsi.kalpala@pfizer.com | **Clinical Groups (FinnGen phases 1&2)** | **Gastroenterology Group** |
| Melissa Miller | Pfizer, New York, NY, United States | melissa.r.miller@pfizer.com | **Clinical Groups (FinnGen phases 1&2)** | **Gastroenterology Group** |
| Xinli Hu | Pfizer, New York, NY, United States | xinli.hu@pfizer.com | **Clinical Groups (FinnGen phases 1&2)** | **Gastroenterology Group** |
| Kari Eklund | Hospital District of Helsinki and Uusimaa, Helsinki, Finland | kari.eklund@hus.fi | **Clinical Groups (FinnGen phases 1&2)** | **Rheumatology Group** |
| Antti Palomäki | Wellbeing Services County of Southwest Finland, Turku, Finland | ajpalo@utu.fi | **Clinical Groups (FinnGen phases 1&2)** | **Rheumatology Group** |
| Pia Isomäki | Wellbeing Services County of Pirkanmaa, Tampere, Finland | pia.isomaki@pirha.fi | **Clinical Groups (FinnGen phases 1&2)** | **Rheumatology Group** |
| Laura Pirilä | Wellbeing Services County of Southwest Finland, Turku, Finland | laura.pirila@fimnet.fi,laura.pirila@tyks.fi | **Clinical Groups (FinnGen phases 1&2)** | **Rheumatology Group** |
| Oili Kaipiainen-Seppänen | Wellbeing services county of North Savo, Kuopio, Finland | oili.kaipiainen-seppanen@kuh.fi | **Clinical Groups (FinnGen phases 1&2)** | **Rheumatology Group** |
| Johanna Huhtakangas | Wellbeing services county of North Ostrobothnia, Oulu, Finland | johanna.huhtakangas@kuh.fi | **Clinical Groups (FinnGen phases 1&2)** | **Rheumatology Group** |
| Nina Mars | Institute for Molecular Medicine Finland (FIMM), HiLIFE, University of Helsinki, Helsinki, Finland | nina.mars@helsinki.fi | **Clinical Groups (FinnGen phases 1&2)** | **Rheumatology Group** |
| Jeffrey Waring | Abbvie, Chicago, IL, United States | jeff.waring@abbvie.com | **Clinical Groups (FinnGen phases 1&2)** | **Rheumatology Group** |
| Fedik Rahimov | Abbvie, Chicago, IL, United States | fedik.rahimov@abbvie.com | **Clinical Groups (FinnGen phases 1&2)** | **Rheumatology Group** |
| Apinya Lertratanakul | Abbvie, Chicago, IL, United States | apinya.lertratanakul@abbvie.com | **Clinical Groups (FinnGen phases 1&2)** | **Rheumatology Group** |
| Nizar Smaoui | Abbvie, Chicago, IL, United States | nizar.smaoui@abbvie.com | **Clinical Groups (FinnGen phases 1&2)** | **Rheumatology Group** |
| Anne Lehtonen | Abbvie, Chicago, IL, United States | anne.lehtonen@abbvie.com | **Clinical Groups (FinnGen phases 1&2)** | **Rheumatology Group** |
| Coralie Viollet | AstraZeneca, Cambridge, United Kingdom | coralie.viollet@astrazeneca.com | **Clinical Groups (FinnGen phases 1&2)** | **Rheumatology Group** |
| Marla Hochfeld | Bristol Myers Squibb, New York, NY, United States | mhochfeld@celgene.com | **Clinical Groups (FinnGen phases 1&2)** | **Rheumatology Group** |
| Natalie Bowers | Genentech, San Francisco, CA, United States | bowersn1@gene.com | **Clinical Groups (FinnGen phases 1&2)** | **Rheumatology Group** |
| Rion Pendergrass | Genentech, San Francisco, CA, United States | penders2@gene.com | **Clinical Groups (FinnGen phases 1&2)** | **Rheumatology Group** |
| Jorge Esparza Gordillo | GlaxoSmithKline, Brentford, United Kingdom | jorge.x.esparza-gordillo@gsk.com | **Clinical Groups (FinnGen phases 1&2)** | **Rheumatology Group** |
| Dawn Waterworth | Johnson & Johnson Innovative Medicine, Spring House, PA, United States | dwaterwo@its.jnj.com | **Clinical Groups (FinnGen phases 1&2)** | **Rheumatology Group** |
| Fabiana Farias | Merck, Kenilworth, NJ, United States | fabiana.farias@merck.com | **Clinical Groups (FinnGen phases 1&2)** | **Rheumatology Group** |
| Kirsi Kalpala | Pfizer, New York, NY, United States | kirsi.kalpala@pfizer.com | **Clinical Groups (FinnGen phases 1&2)** | **Rheumatology Group** |
| Nan Bing | Pfizer, New York, NY, United States | nan.bing@pfizer.com | **Clinical Groups (FinnGen phases 1&2)** | **Rheumatology Group** |
| Xinli Hu | Pfizer, New York, NY, United States | xinli.hu@pfizer.com | **Clinical Groups (FinnGen phases 1&2)** | **Rheumatology Group** |
| Tarja Laitinen | Wellbeing Services County of Pirkanmaa, Tampere, Finland | tarja.laitinen@pirha.fi | **Clinical Groups (FinnGen phases 1&2)** | **Pulmonology Group** |
| Margit Pelkonen | Wellbeing services county of North Savo, Kuopio, Finland | margit.pelkonen@kuh.fi | **Clinical Groups (FinnGen phases 1&2)** | **Pulmonology Group** |
| Paula Kauppi | Hospital District of Helsinki and Uusimaa, Helsinki, Finland | paula.kauppi@hus.fi | **Clinical Groups (FinnGen phases 1&2)** | **Pulmonology Group** |
| Hannu Kankaanranta | University of Gothenburg, Gothenburg, Sweden/ Seinäjoki Central Hospital, Seinäjoki, Finland/ Tampere University, Tampere, Finland | hannu.kankaanranta@tuni.fi | **Clinical Groups (FinnGen phases 1&2)** | **Pulmonology Group** |
| Terttu Harju | Wellbeing services county of North Ostrobothnia, Oulu, Finland | terttu.harju@oulu.fi | **Clinical Groups (FinnGen phases 1&2)** | **Pulmonology Group** |
| Riitta Lahesmaa | Wellbeing Services County of Southwest Finland, Turku, Finland | rilahes@utu.fi | **Clinical Groups (FinnGen phases 1&2)** | **Pulmonology Group** |
| Nizar Smaoui | Abbvie, Chicago, IL, United States | nizar.smaoui@abbvie.com | **Clinical Groups (FinnGen phases 1&2)** | **Pulmonology Group** |
| Coralie Viollet | AstraZeneca, Cambridge, United Kingdom | coralie.viollet@astrazeneca.com | **Clinical Groups (FinnGen phases 1&2)** | **Pulmonology Group** |
| Susan Eaton | Biogen, Cambridge, MA, United States | susan.eaton@biogen.com | **Clinical Groups (FinnGen phases 1&2)** | **Pulmonology Group** |
| Hubert Chen | Genentech, San Francisco, CA, United States | chenh37@gene.com | **Clinical Groups (FinnGen phases 1&2)** | **Pulmonology Group** |
| Rion Pendergrass | Genentech, San Francisco, CA, United States | penders2@gene.com | **Clinical Groups (FinnGen phases 1&2)** | **Pulmonology Group** |
| Natalie Bowers | Genentech, San Francisco, CA, United States | bowersn1@gene.com | **Clinical Groups (FinnGen phases 1&2)** | **Pulmonology Group** |
| Joanna Betts | GlaxoSmithKline, Brentford, United Kingdom | joanna.c.betts@gsk.com | **Clinical Groups (FinnGen phases 1&2)** | **Pulmonology Group** |
| Kirsi Auro | GlaxoSmithKline, Espoo, Finland | kirsi.m.auro@gsk.com | **Clinical Groups (FinnGen phases 1&2)** | **Pulmonology Group** |
| Rajashree Mishra | GlaxoSmithKline, Brentford, United Kingdom | rajashree.x.mishra@gsk.com | **Clinical Groups (FinnGen phases 1&2)** | **Pulmonology Group** |
| Majd Mouded | Novartis, Basel, Switzerland | majd.mouded@novartis.com | **Clinical Groups (FinnGen phases 1&2)** | **Pulmonology Group** |
| Debby Ngo | Novartis, Basel, Switzerland | debby.ngo@novartis.com | **Clinical Groups (FinnGen phases 1&2)** | **Pulmonology Group** |
| Teemu Niiranen | University of Turku, Turku, Finland; Finnish Institute for Health and Welfare (THL), Helsinki, Finland | teemu.niiranen@thl.fi | **Clinical Groups (FinnGen phases 1&2)** | **Cardiometabolic Diseases Group** |
| Felix Vaura | Finnish Institute for Health and Welfare (THL), Helsinki, Finland | fechva@utu.fi | **Clinical Groups (FinnGen phases 1&2)** | **Cardiometabolic Diseases Group** |
| Veikko Salomaa | Finnish Institute for Health and Welfare (THL), Helsinki, Finland | veikko.salomaa@thl.fi | **Clinical Groups (FinnGen phases 1&2)** | **Cardiometabolic Diseases Group** |
| Kaj Metsärinne | Wellbeing Services County of Southwest Finland, Turku, Finland | kaj.metsarinne@tyks.fi | **Clinical Groups (FinnGen phases 1&2)** | **Cardiometabolic Diseases Group** |
| Jenni Aittokallio | Wellbeing Services County of Southwest Finland, Turku, Finland | jemato@utu.fi | **Clinical Groups (FinnGen phases 1&2)** | **Cardiometabolic Diseases Group** |
| Mika Kähönen | Finnish Clinical Biobank Tampere / University of Tampere / Wellbeing Services County of Pirkanmaa, Tampere, Finland | mika.kahonen@uta.fi | **Clinical Groups (FinnGen phases 1&2)** | **Cardiometabolic Diseases Group** |
| Jussi Hernesniemi | Wellbeing Services County of Pirkanmaa, Tampere, Finland | jussi.hernesniemi@tuni.fi | **Clinical Groups (FinnGen phases 1&2)** | **Cardiometabolic Diseases Group** |
| Daniel Gordin | Hospital District of Helsinki and Uusimaa, Helsinki, Finland | daniel.gordin@hus.fi | **Clinical Groups (FinnGen phases 1&2)** | **Cardiometabolic Diseases Group** |
| Juha Sinisalo | Hospital District of Helsinki and Uusimaa, Helsinki, Finland | juha.sinisalo@hus.fi | **Clinical Groups (FinnGen phases 1&2)** | **Cardiometabolic Diseases Group** |
| Marja-Riitta Taskinen | Hospital District of Helsinki and Uusimaa, Helsinki, Finland | marja-riitta.taskinen@helsinki.fi | **Clinical Groups (FinnGen phases 1&2)** | **Cardiometabolic Diseases Group** |
| Tiinamaija Tuomi | Institute for Molecular Medicine Finland (FIMM), HiLIFE, University of Helsinki, Helsinki, Finland; Hospital District of Helsinki and Uusimaa, Helsinki, Finland | tiinamaija.tuomi@hus.fi | **Clinical Groups (FinnGen phases 1&2)** | **Cardiometabolic Diseases Group** |
| Timo Hiltunen | Hospital District of Helsinki and Uusimaa, Helsinki, Finland | timo.hiltunen@hus.fi | **Clinical Groups (FinnGen phases 1&2)** | **Cardiometabolic Diseases Group** |
| Jari Laukkanen | Central Finland Biobank / University of Jyväskylä / Wellbeing Services County of Central Finland, Jyväskylä, Finland | jari.laukkanen@hyvaks.fi | **Clinical Groups (FinnGen phases 1&2)** | **Cardiometabolic Diseases Group** |
| Amanda Elliott | Institute for Molecular Medicine Finland (FIMM), HiLIFE, University of Helsinki, Helsinki, Finland; Broad Institute, Cambridge, MA, USA and Massachusetts General Hospital, Boston, MA, USA | aelliott@broadinstitute.org | **Clinical Groups (FinnGen phases 1&2)** | **Cardiometabolic Diseases Group** |
| Mary Pat Reeve | Institute for Molecular Medicine Finland (FIMM), HiLIFE, University of Helsinki, Helsinki, Finland | mary.reeve@helsinki.fi | **Clinical Groups (FinnGen phases 1&2)** | **Cardiometabolic Diseases Group** |
| Sanni Ruotsalainen | Institute for Molecular Medicine Finland (FIMM), HiLIFE, University of Helsinki, Helsinki, Finland | sanni.ruotsalainen@helsinki.fi | **Clinical Groups (FinnGen phases 1&2)** | **Cardiometabolic Diseases Group** |
| Dirk Paul | Astra Zeneca, Cambridge, United Kingdom | dirk.paul@astrazeneca.com | **Clinical Groups (FinnGen phases 1&2)** | **Cardiometabolic Diseases Group** |
| Natalie Bowers | Genentech, San Francisco, CA, United States | bowersn1@gene.com | **Clinical Groups (FinnGen phases 1&2)** | **Cardiometabolic Diseases Group** |
| Rion Pendergrass | Genentech, San Francisco, CA, United States | penders2@gene.com | **Clinical Groups (FinnGen phases 1&2)** | **Cardiometabolic Diseases Group** |
| Audrey Chu | GlaxoSmithKline, Brentford, United Kingdom | audrey.y.chu@gsk.com | **Clinical Groups (FinnGen phases 1&2)** | **Cardiometabolic Diseases Group** |
| Dermot Reilly | Johnson & Johnson Innovative Medicine, Boston, MA, United States | dreill11@its.jnj.com | **Clinical Groups (FinnGen phases 1&2)** | **Cardiometabolic Diseases Group** |
| Mike Mendelson | Novartis, Boston, MA, United States | mike.mendelson@novartis.com | **Clinical Groups (FinnGen phases 1&2)** | **Cardiometabolic Diseases Group** |
| Jaakko Parkkinen | Pfizer, New York, NY, United States | jaakko.parkkinen@pfizer.com | **Clinical Groups (FinnGen phases 1&2)** | **Cardiometabolic Diseases Group** |
| Melissa Miller | Pfizer, New York, NY, United States | melissa.r.miller@pfizer.com | **Clinical Groups (FinnGen phases 1&2)** | **Cardiometabolic Diseases Group** |
| Tuomo Meretoja | Helsinki University Hospital and University of Helsinki, Helsinki, Finland | tuomo.meretoja@hus.fi | **Clinical Groups (FinnGen phases 1&2)** | **Oncology Group** |
| Heikki Joensuu | Helsinki University Hospital and University of Helsinki, Helsinki, Finland | heikki.joensuu@hus.fi | **Clinical Groups (FinnGen phases 1&2)** | **Oncology Group** |
| Olli Carpén | Hospital District of Helsinki and Uusimaa, Helsinki, Finland | olli.carpen@helsinki.fi | **Clinical Groups (FinnGen phases 1&2)** | **Oncology Group** |
| Johanna Mattson | Hospital District of Helsinki and Uusimaa, Helsinki, Finland | johanna.mattson@hus.fi | **Clinical Groups (FinnGen phases 1&2)** | **Oncology Group** |
| Eveliina Salminen | Hospital District of Helsinki and Uusimaa, Helsinki, Finland | eveliina.e.salminen@hus.fi | **Clinical Groups (FinnGen phases 1&2)** | **Oncology Group** |
| Annika Auranen | Wellbeing Services County of Pirkanmaa, Tampere, Finland | anaura@utu.fi | **Clinical Groups (FinnGen phases 1&2)** | **Oncology Group** |
| Peeter Karihtala | Helsinki University Hospital and University of Helsinki, Helsinki, Finland | peeter.karihtala@hus.fi | **Clinical Groups (FinnGen phases 1&2)** | **Oncology Group** |
| Päivi Auvinen | Wellbeing services county of North Savo, Kuopio, Finland | paivi.auvinen@kuh.fi | **Clinical Groups (FinnGen phases 1&2)** | **Oncology Group** |
| Klaus Elenius | Wellbeing Services County of Southwest Finland, Turku, Finland | klaus.elenius@utu.fi | **Clinical Groups (FinnGen phases 1&2)** | **Oncology Group** |
| Johanna Schleutker | Wellbeing Services County of Southwest Finland, Turku, Finland | johanna.schleutker@utu.fi | **Clinical Groups (FinnGen phases 1&2)** | **Oncology Group** |
| Esa Pitkänen | Institute for Molecular Medicine Finland (FIMM), HiLIFE, University of Helsinki, Helsinki, Finland | esa.pitkanen@helsinki.fi | **Clinical Groups (FinnGen phases 1&2)** | **Oncology Group** |
| Nina Mars | Institute for Molecular Medicine Finland (FIMM), HiLIFE, University of Helsinki, Helsinki, Finland | nina.mars@helsinki.fi | **Clinical Groups (FinnGen phases 1&2)** | **Oncology Group** |
| Mark Daly | Institute for Molecular Medicine Finland (FIMM), HiLIFE, University of Helsinki, Helsinki, Finland; Broad Institute of MIT and Harvard; Massachusetts General Hospital, Boston, MA, United States | mark.daly@helsinki.fi | **Clinical Groups (FinnGen phases 1&2)** | **Oncology Group** |
| Relja Popovic | Abbvie, Chicago, IL, United States | relja.popovic@abbvie.com | **Clinical Groups (FinnGen phases 1&2)** | **Oncology Group** |
| Jeffrey Waring | Abbvie, Chicago, IL, United States | jeff.waring@abbvie.com | **Clinical Groups (FinnGen phases 1&2)** | **Oncology Group** |
| Bridget Riley-Gillis | Abbvie, Chicago, IL, United States | bridget.rileygillis@abbvie.com | **Clinical Groups (FinnGen phases 1&2)** | **Oncology Group** |
| Anne Lehtonen | Abbvie, Chicago, IL, United States | anne.lehtonen@abbvie.com | **Clinical Groups (FinnGen phases 1&2)** | **Oncology Group** |
| Margarete Fabre | AstraZeneca, Cambridge, United Kingdom | margarete.fabre@astrazeneca.com | **Clinical Groups (FinnGen phases 1&2)** | **Oncology Group** |
| Jennifer Schutzman | Genentech, San Francisco, CA, United States | schutzman.jennifer@gene.com | **Clinical Groups (FinnGen phases 1&2)** | **Oncology Group** |
| Natalie Bowers | Genentech, San Francisco, CA, United States | bowersn1@gene.com | **Clinical Groups (FinnGen phases 1&2)** | **Oncology Group** |
| Rion Pendergrass | Genentech, San Francisco, CA, United States | penders2@gene.com | **Clinical Groups (FinnGen phases 1&2)** | **Oncology Group** |
| Diptee Kulkarni | GlaxoSmithKline, Brentford, United Kingdom | diptee.a.kulkarni@gsk.com | **Clinical Groups (FinnGen phases 1&2)** | **Oncology Group** |
| Alessandro Porello | Johnson & Johnson Innovative Medicine, Spring House, PA, United States | APorrell@ITS.JNJ.com | **Clinical Groups (FinnGen phases 1&2)** | **Oncology Group** |
| Andrey Loboda | Merck, Kenilworth, NJ, United States | andrey_loboda@merck.com | **Clinical Groups (FinnGen phases 1&2)** | **Oncology Group** |
| Stefan McDonough | Pfizer, New York, NY, United States | stefan.McDonough@pfizer.com | **Clinical Groups (FinnGen phases 1&2)** | **Oncology Group** |
| Kai Kaarniranta | Wellbeing services county of North Savo, Kuopio, Finland; University of Lodz, Lodz, Poland | kai.kaarniranta@uef.fi | **Clinical Groups (FinnGen phases 1&2)** | **Opthalmology Group** |
| Joni A Turunen | Helsinki University Hospital and University of Helsinki, Helsinki, Finland; Folkhälsan Research Center, Helsinki, Finland | joni.turunen@helsinki.fi | **Clinical Groups (FinnGen phases 1&2)** | **Opthalmology Group** |
| Terhi Ollila | Hospital District of Helsinki and Uusimaa, Helsinki, Finland | terhi.ollila@hus.fi | **Clinical Groups (FinnGen phases 1&2)** | **Opthalmology Group** |
| Hannu Uusitalo | Wellbeing Services County of Pirkanmaa, Tampere, Finland | hannu.uusitalo@tuni.fi | **Clinical Groups (FinnGen phases 1&2)** | **Opthalmology Group** |
| Juha Karjalainen | Institute for Molecular Medicine Finland (FIMM), HiLIFE, University of Helsinki, Helsinki, Finland | juha.karjalainen@helsinki.fi | **Clinical Groups (FinnGen phases 1&2)** | **Opthalmology Group** |
| Esa Pitkänen | Institute for Molecular Medicine Finland (FIMM), HiLIFE, University of Helsinki, Helsinki, Finland | esa.pitkanen@helsinki.fi | **Clinical Groups (FinnGen phases 1&2)** | **Opthalmology Group** |
| Mengzhen Liu | Abbvie, Chicago, IL, United States | mengzhen.liu@abbvie.com | **Clinical Groups (FinnGen phases 1&2)** | **Opthalmology Group** |
| Erich Strauss | Genentech, San Francisco, CA, United States | strauss.erich@gene.com | **Clinical Groups (FinnGen phases 1&2)** | **Opthalmology Group** |
| Natalie Bowers | Genentech, San Francisco, CA, United States | bowersn1@gene.com | **Clinical Groups (FinnGen phases 1&2)** | **Opthalmology Group** |
| Hao Chen | Genentech, San Francisco, CA, United States | haoc@gene.com | **Clinical Groups (FinnGen phases 1&2)** | **Opthalmology Group** |
| Rion Pendergrass | Genentech, San Francisco, CA, United States | penders2@gene.com | **Clinical Groups (FinnGen phases 1&2)** | **Opthalmology Group** |
| Kaisa Tasanen | Wellbeing services county of North Ostrobothnia, Oulu, Finland | kaisa.tasanen-maatta@oulu.fi | **Clinical Groups (FinnGen phases 1&2)** | **Dermatology Group** |
| Laura Huilaja | Wellbeing services county of North Ostrobothnia, Oulu, Finland | laura.huilaja@oulu.fi | **Clinical Groups (FinnGen phases 1&2)** | **Dermatology Group** |
| Katariina Hannula-Jouppi | Hospital District of Helsinki and Uusimaa, Helsinki, Finland | katariina.hannula-jouppi@hus.fi | **Clinical Groups (FinnGen phases 1&2)** | **Dermatology Group** |
| Teea Salmi | Wellbeing Services County of Pirkanmaa, Tampere, Finland | teea.salmi@tuni.fi | **Clinical Groups (FinnGen phases 1&2)** | **Dermatology Group** |
| Sirkku Peltonen | Wellbeing Services County of Southwest Finland, Turku, Finland | sipelto@utu.fi | **Clinical Groups (FinnGen phases 1&2)** | **Dermatology Group** |
| Leena Koulu | Wellbeing Services County of Southwest Finland, Turku, Finland | leena.koulu@tyks.fi | **Clinical Groups (FinnGen phases 1&2)** | **Dermatology Group** |
| Nizar Smaoui | Abbvie, Chicago, IL, United States | nizar.smaoui@abbvie.com | **Clinical Groups (FinnGen phases 1&2)** | **Dermatology Group** |
| Fedik Rahimov | Abbvie, Chicago, IL, United States | fedik.rahimov@abbvie.com | **Clinical Groups (FinnGen phases 1&2)** | **Dermatology Group** |
| Anne Lehtonen | Abbvie, Chicago, IL, United States | anne.lehtonen@abbvie.com | **Clinical Groups (FinnGen phases 1&2)** | **Dermatology Group** |
| David Choy | Genentech, San Francisco, CA, United States | choy.david@gene.com | **Clinical Groups (FinnGen phases 1&2)** | **Dermatology Group** |
| Rion Pendergrass | Genentech, San Francisco, CA, United States | penders2@gene.com | **Clinical Groups (FinnGen phases 1&2)** | **Dermatology Group** |
| Dawn Waterworth | Johnson & Johnson Innovative Medicine, Spring House, PA, United States | dwaterwo@its.jnj.com | **Clinical Groups (FinnGen phases 1&2)** | **Dermatology Group** |
| Kirsi Kalpala | Pfizer, New York, NY, United States | kirsi.kalpala@pfizer.com | **Clinical Groups (FinnGen phases 1&2)** | **Dermatology Group** |
| Ying Wu | Pfizer, New York, NY, United States | ying.wu3@pfizer.com | **Clinical Groups (FinnGen phases 1&2)** | **Dermatology Group** |
| Pirkko Pussinen | Hospital District of Helsinki and Uusimaa, Helsinki, Finland | pirkko.pussinen@helsinki.fi | **Clinical Groups (FinnGen phases 1&2)** | **Odontology Group** |
| Aino Salminen | Hospital District of Helsinki and Uusimaa, Helsinki, Finland | aino.m.salminen@helsinki.fi | **Clinical Groups (FinnGen phases 1&2)** | **Odontology Group** |
| Tuula Salo | Hospital District of Helsinki and Uusimaa, Helsinki, Finland | tuula.salo@helsinki.fi | **Clinical Groups (FinnGen phases 1&2)** | **Odontology Group** |
| David Rice | Hospital District of Helsinki and Uusimaa, Helsinki, Finland | david.rice@helsinki.fi | **Clinical Groups (FinnGen phases 1&2)** | **Odontology Group** |
| Pekka Nieminen | Hospital District of Helsinki and Uusimaa, Helsinki, Finland | pekka.nieminen@helsinki.fi | **Clinical Groups (FinnGen phases 1&2)** | **Odontology Group** |
| Ulla Palotie | Hospital District of Helsinki and Uusimaa, Helsinki, Finland | ulla.palotie@helsinki.fi | **Clinical Groups (FinnGen phases 1&2)** | **Odontology Group** |
| Maria Siponen | Wellbeing services county of North Savo, Kuopio, Finland | maria.siponen@uef.fi | **Clinical Groups (FinnGen phases 1&2)** | **Odontology Group** |
| Liisa Suominen | Wellbeing services county of North Savo, Kuopio, Finland | liisa.suominen@uef.fi | **Clinical Groups (FinnGen phases 1&2)** | **Odontology Group** |
| Päivi Mäntylä | Wellbeing services county of North Savo, Kuopio, Finland | paivi.mantyla@uef.fi | **Clinical Groups (FinnGen phases 1&2)** | **Odontology Group** |
| Ulvi Gursoy | Wellbeing Services County of Southwest Finland, Turku, Finland | ulvi.gursoy@utu.fi | **Clinical Groups (FinnGen phases 1&2)** | **Odontology Group** |
| Vuokko Anttonen | Wellbeing services county of North Ostrobothnia, Oulu, Finland | vuokko.anttonen@oulu.fi | **Clinical Groups (FinnGen phases 1&2)** | **Odontology Group** |
| Kirsi Sipilä | Oulu University Hospital and University of Oulu, Oulu, Finland | kirsi.sipila@oulu.fi | **Clinical Groups (FinnGen phases 1&2)** | **Odontology Group** |
| Rion Pendergrass | Genentech, San Francisco, CA, United States | pendergrass.sarah@gene.com | **Clinical Groups (FinnGen phases 1&2)** | **Odontology Group** |
| Hannele Laivuori | Institute for Molecular Medicine Finland (FIMM), HiLIFE, University of Helsinki, Helsinki, Finland | hannele.laivuori@helsinki.fi | **Clinical Groups (FinnGen phases 1&2)** | **Women’s Health and Reproduction Group** |
| Venla Kurra | Wellbeing Services County of Pirkanmaa, Tampere, Finland | venla.kurra@tuni.fi | **Clinical Groups (FinnGen phases 1&2)** | **Women’s Health and Reproduction Group** |
| Laura Kotaniemi-Talonen | Wellbeing Services County of Pirkanmaa, Tampere, Finland | laura.kotaniemi-talonen@tuni.fi | **Clinical Groups (FinnGen phases 1&2)** | **Women’s Health and Reproduction Group** |
| Oskari Heikinheimo | Hospital District of Helsinki and Uusimaa, Helsinki, Finland | oskari.heikinheimo@helsinki.fi | **Clinical Groups (FinnGen phases 1&2)** | **Women’s Health and Reproduction Group** |
| Ilkka Kalliala | Hospital District of Helsinki and Uusimaa, Helsinki, Finland | ilkka.kalliala@hus.fi | **Clinical Groups (FinnGen phases 1&2)** | **Women’s Health and Reproduction Group** |
| Lauri Aaltonen | Hospital District of Helsinki and Uusimaa, Helsinki, Finland | lauri.aaltonen@helsinki.fi | **Clinical Groups (FinnGen phases 1&2)** | **Women’s Health and Reproduction Group** |
| Varpu Jokimaa | Wellbeing Services County of Southwest Finland, Turku, Finland | varpu.jokimaa@utu.fi | **Clinical Groups (FinnGen phases 1&2)** | **Women’s Health and Reproduction Group** |
| Johannes Kettunen | Northern Finland Biobank Borealis / University of Oulu / Wellbeing services county of North Ostrobothnia, Oulu, Finland | Johannes.Kettunen@oulu.fi | **Clinical Groups (FinnGen phases 1&2)** | **Women’s Health and Reproduction Group** |
| Marja Vääräsmäki | Wellbeing services county of North Ostrobothnia, Oulu, Finland | marja.vaarasmaki@oulu.fi | **Clinical Groups (FinnGen phases 1&2)** | **Women’s Health and Reproduction Group** |
| Outi Uimari | Wellbeing services county of North Ostrobothnia, Oulu, Finland | outi.uimari@oulu.fi | **Clinical Groups (FinnGen phases 1&2)** | **Women’s Health and Reproduction Group** |
| Laure Morin-Papunen | Wellbeing services county of North Ostrobothnia, Oulu, Finland | lmp@cc.oulu.fi | **Clinical Groups (FinnGen phases 1&2)** | **Women’s Health and Reproduction Group** |
| Maarit Niinimäki | Wellbeing services county of North Ostrobothnia, Oulu, Finland | maarit.niinimaki@oulu.fi | **Clinical Groups (FinnGen phases 1&2)** | **Women’s Health and Reproduction Group** |
| Terhi Piltonen | Wellbeing services county of North Ostrobothnia, Oulu, Finland | terhi.piltonen@oulu.fi | **Clinical Groups (FinnGen phases 1&2)** | **Women’s Health and Reproduction Group** |
| Katja Kivinen | Institute for Molecular Medicine Finland (FIMM), HiLIFE, University of Helsinki, Helsinki, Finland | katja.kivinen@helsinki.fi | **Clinical Groups (FinnGen phases 1&2)** | **Women’s Health and Reproduction Group** |
| Elisabeth Widen | Institute for Molecular Medicine Finland (FIMM), HiLIFE, University of Helsinki, Helsinki, Finland | elisabeth.widen@helsinki.fi | **Clinical Groups (FinnGen phases 1&2)** | **Women’s Health and Reproduction Group** |
| Taru Tukiainen | Institute for Molecular Medicine Finland (FIMM), HiLIFE, University of Helsinki, Helsinki, Finland | taru.tukiainen@helsinki.fi | **Clinical Groups (FinnGen phases 1&2)** | **Women’s Health and Reproduction Group** |
| Mary Pat Reeve | Institute for Molecular Medicine Finland (FIMM), HiLIFE, University of Helsinki, Helsinki, Finland | mary.reeve@helsinki.fi | **Clinical Groups (FinnGen phases 1&2)** | **Women’s Health and Reproduction Group** |
| Mark Daly | Institute for Molecular Medicine Finland (FIMM), HiLIFE, University of Helsinki, Helsinki, Finland; Broad Institute of MIT and Harvard; Massachusetts General Hospital, Boston, MA, United States | mark.daly@helsinki.fi | **Clinical Groups (FinnGen phases 1&2)** | **Women’s Health and Reproduction Group** |
| Niko Välimäki | University of Helsinki, Helsinki, Finland | niko.valimaki@helsinki.fi | **Clinical Groups (FinnGen phases 1&2)** | **Women’s Health and Reproduction Group** |
| Eija Laakkonen | University of Jyväskylä, Jyväskylä, Finland | eija.k.laakkonen@jyu.fi | **Clinical Groups (FinnGen phases 1&2)** | **Women’s Health and Reproduction Group** |
| Jaakko Tyrmi | University of Oulu, Oulu, Finland / University of Tampere, Tampere, Finland | jaakko.tyrmi@oulu.fi | **Clinical Groups (FinnGen phases 1&2)** | **Women’s Health and Reproduction Group** |
| Heidi Silven | University of Oulu, Oulu, Finland | heidi.silven@student.oulu.fi | **Clinical Groups (FinnGen phases 1&2)** | **Women’s Health and Reproduction Group** |
| Eeva Sliz | University of Oulu, Oulu, Finland | eeva.sliz@oulu.fi | **Clinical Groups (FinnGen phases 1&2)** | **Women’s Health and Reproduction Group** |
| Riikka Arffman | University of Oulu, Oulu, Finland | riikka.arffman@oulu.fi | **Clinical Groups (FinnGen phases 1&2)** | **Women’s Health and Reproduction Group** |
| Susanna Savukoski | University of Oulu, Oulu, Finland | susanna.savukoski@oulu.fi | **Clinical Groups (FinnGen phases 1&2)** | **Women’s Health and Reproduction Group** |
| Triin Laisk | Estonian biobank, Tartu, Estonia | triin.laisk@ut.ee | **Clinical Groups (FinnGen phases 1&2)** | **Women’s Health and Reproduction Group** |
| Natalia Pujol | Estonian biobank, Tartu, Estonia | natalia.pujolgualdo@oulu.fi | **Clinical Groups (FinnGen phases 1&2)** | **Women’s Health and Reproduction Group** |
| Mengzhen Liu | AbbVie, Chicago, IL, United States | mengzhen.liu@abbvie.com | **Clinical Groups (FinnGen phases 1&2)** | **Women’s Health and Reproduction Group** |
| Bridget Riley-Gillis | AbbVie, Chicago, IL, United States | bridget.rileygillis@abbvie.com | **Clinical Groups (FinnGen phases 1&2)** | **Women’s Health and Reproduction Group** |
| Rion Pendergrass | Genentech, San Francisco, CA, United States | penders2@gene.com | **Clinical Groups (FinnGen phases 1&2)** | **Women’s Health and Reproduction Group** |
| Janet Kumar | GlaxoSmithKline, Collegeville, PA, United States | janet.x.kumar@gsk.com | **Clinical Groups (FinnGen phases 1&2)** | **Women’s Health and Reproduction Group** |
| Iiris Hovatta | University of Helsinki, Finland | iiris.hovatta@helsinki.fi | **Clinical Groups (FinnGen phases 1&2)** | **Depression group** |
| Erkki Isometsä | Hospital District of Helsinki and Uusimaa, Helsinki, Finland | erkki.isometsa@hus.fi | **Clinical Groups (FinnGen phases 1&2)** | **Depression group** |
| Hanna Ollila | Institute for Molecular Medicine Finland (FIMM), HiLIFE, University of Helsinki, Helsinki, Finland | hanna.m.ollila@helsinki.fi | **Clinical Groups (FinnGen phases 1&2)** | **Depression group** |
| Jaana Suvisaari | Finnish Institute for Health and Welfare (THL), Helsinki, Finland | jaana.suvisaari@thl.fi | **Clinical Groups (FinnGen phases 1&2)** | **Depression group** |
| Antti Mäkitie | University of Helsinki and Helsinki University Hospital, Helsinki, Finland | antti.makitie@helsinki.fi | **Clinical Groups (FinnGen phases 1&2)** | **ENT (ear, nose and throath) Group** |
| Argyro Bizaki-Vallaskangas | Wellbeing Services County of Pirkanmaa, Tampere, Finland | argyro.bizaki-vallaskangas@tuni.fi | **Clinical Groups (FinnGen phases 1&2)** | **ENT (ear, nose and throath) Group** |
| Sanna Toppila-Salmi | University of Eastern Finland and Kuopio University Hospital, Kuopio, Finland; Helsinki University Hospital and University of Helsinki, Finland | sanna.salmi@helsinki.fi | **Clinical Groups (FinnGen phases 1&2)** | **ENT (ear, nose and throath) Group** |
| Tytti Willberg | Wellbeing Services County of Southwest Finland, Turku, Finland | tytti.willberg@tyks.fi | **Clinical Groups (FinnGen phases 1&2)** | **ENT (ear, nose and throath) Group** |
| Elmo Saarentaus | Institute for Molecular Medicine Finland (FIMM), HiLIFE, University of Helsinki, Helsinki, Finland | elmo.saarentaus@helsinki.fi | **Clinical Groups (FinnGen phases 1&2)** | **ENT (ear, nose and throath) Group** |
| Antti Aarnisalo | Hospital District of Helsinki and Uusimaa, Helsinki, Finland | antti.aarnisalo@hus.fi | **Clinical Groups (FinnGen phases 1&2)** | **ENT (ear, nose and throath) Group** |
| Eveliina Salminen | Hospital District of Helsinki and Uusimaa, Helsinki, Finland | eveliina.e.salminen@hus.fi | **Clinical Groups (FinnGen phases 1&2)** | **ENT (ear, nose and throath) Group** |
| Elisa Rahikkala | Northern Ostrobothnia Hospital District, Oulu, Finland | elisa.rahikkala@oulu.fi | **Clinical Groups (FinnGen phases 1&2)** | **ENT (ear, nose and throath) Group** |
| Johannes Kettunen | Northern Finland Biobank Borealis / University of Oulu / Wellbeing services county of North Ostrobothnia, Oulu, Finland | johannes.kettunen@oulu.fi | **Clinical Groups (FinnGen phases 1&2)** | **ENT (ear, nose and throath) Group** |
| Kristiina Aittomäki | Helsinki University Central Hospital, Helsinki, Finland | kristiina.aittomaki@helsinki.fi | **Clinical Groups (FinnGen phases 1&2)** | **POI (premature ovarian failure) Group** |
| Fredrik Åberg | Helsinki University Hospital and University of Helsinki, Helsinki, Finland | fredrik.aberg@helsinki.fi | **Clinical Groups (FinnGen phases 1&2)** | **LiverScore Group** |
| Joel Rämö | Institute for Molecular Medicine Finland (FIMM), HiLIFE, University of Helsinki, Helsinki, Finland; Broad Institute, Cambridge, MA, United States | joel.ramo@helsinki.fi | **Clinical Task Forces (FinnGen phase 3)** | **Eye diseases Task Force** |
| Mark Daly | Institute for Molecular Medicine Finland (FIMM), HiLIFE, University of Helsinki, Helsinki, Finland; Broad Institute of MIT and Harvard; Massachusetts General Hospital, Boston, MA, United States | mjdaly@broadinstitute.org | **Clinical Task Forces (FinnGen phase 3)** | **Eye diseases Task Force** |
| Mary Pat Reeve | Institute for Molecular Medicine Finland (FIMM), HiLIFE, University of Helsinki, Helsinki, Finland; Broad Institute, Cambridge, MA, United States | mary.reeve@helsinki.fi | **Clinical Task Forces (FinnGen phase 3)** | **Eye diseases Task Force** |
| Muhammad Adnan Khan | Institute for Molecular Medicine Finland (FIMM), HiLIFE, University of Helsinki, Helsinki, Finland | adnan.khan@helsinki.fi | **Clinical Task Forces (FinnGen phase 3)** | **Eye diseases Task Force** |
| Johanna Mäkelä | Finnish Biobank Cooperative - FINBB | johanna.makela@finbb.fi | **Clinical Task Forces (FinnGen phase 3)** | **Eye diseases Task Force** |
| Ilkka Immonen | Hospital District of Helsinki and Uusimaa, Helsinki, Finland | ilkka.immonen@hus.fi | **Clinical Task Forces (FinnGen phase 3)** | **Eye diseases Task Force** |
| Kai Kaarniranta | Wellbeing services county of North Savo, Kuopio, Finland; University of Lodz, Lodz, Poland | kai.kaarniranta@uef.fi | **Clinical Task Forces (FinnGen phase 3)** | **Eye diseases Task Force** |
| Joni A Turunen | Helsinki University Hospital and University of Helsinki, Helsinki, Finland; Folkhälsan Research Center, Helsinki, Finland | Joni.Turunen@hus.fi | **Clinical Task Forces (FinnGen phase 3)** | **Eye diseases Task Force** |
| Anneke Den Hollander | AbbVie, Chicago, IL, United States | anneke.denhollander@abbvie.com | **Clinical Task Forces (FinnGen phase 3)** | **Eye diseases Task Force** |
| Bridget Riley-Gillis | AbbVie, Chicago, IL, United States | bridget.rileygillis@abbvie.com | **Clinical Task Forces (FinnGen phase 3)** | **Eye diseases Task Force** |
| Mengzhen Liu | AbbVie, Chicago, IL, United States | mengzhen.liu@abbvie.com | **Clinical Task Forces (FinnGen phase 3)** | **Eye diseases Task Force** |
| Nizar Smaoui | AbbVie, Chicago, IL, United States | nizar.smaoui@abbvie.com | **Clinical Task Forces (FinnGen phase 3)** | **Eye diseases Task Force** |
| Fabio Baschiera | Bayer AG, Leverkusen, Germany | fabio.baschiera@bayer.com | **Clinical Task Forces (FinnGen phase 3)** | **Eye diseases Task Force** |
| Hans van Leeuwen | Bayer AG, Leverkusen, Germany | hans.vanleeuwen@bayer.com | **Clinical Task Forces (FinnGen phase 3)** | **Eye diseases Task Force** |
| Elke Markert | Boehringer Ingelheim, Ingelheim am Rhein, Germany | elke.markert@boehringer-ingelheim.com | **Clinical Task Forces (FinnGen phase 3)** | **Eye diseases Task Force** |
| Brian Yaspan | Genentech, San Francisco, CA, United States | yaspan.brian@gene.com | **Clinical Task Forces (FinnGen phase 3)** | **Eye diseases Task Force** |
| Charli Harlow | GlaxoSmithKline, Collegeville, PA, United States | charli.e.harlow@gsk.com | **Clinical Task Forces (FinnGen phase 3)** | **Eye diseases Task Force** |
| Lea Sarow-Blat | GlaxoSmithKline, Collegeville, PA, United States | lea.2.sarov-blat@gsk.com | **Clinical Task Forces (FinnGen phase 3)** | **Eye diseases Task Force** |
| Dermont Reilly | Johnson & Johnson Innovative Medicine, Spring House, PA, United States | DReill11@its.jnj.com | **Clinical Task Forces (FinnGen phase 3)** | **Eye diseases Task Force** |
| P. Dunnmon | Johnson & Johnson Innovative Medicine, Spring House, PA, United States | PDunnmon@ITS.JNJ.com | **Clinical Task Forces (FinnGen phase 3)** | **Eye diseases Task Force** |
| Sara Gale | Johnson & Johnson Innovative Medicine, Spring House, PA, United States | sgale1@its.jnj.com | **Clinical Task Forces (FinnGen phase 3)** | **Eye diseases Task Force** |
| Fabiana Farias | Merck, Kenilworth, NJ, United States | fabiana.farias@merck.com | **Clinical Task Forces (FinnGen phase 3)** | **Eye diseases Task Force** |
| Jorge Del-aguila | Merck, Kenilworth, NJ, United States | jorge.del-aguila@merck.com | **Clinical Task Forces (FinnGen phase 3)** | **Eye diseases Task Force** |
| Catherine O’Riordan | Translational Sciences, Sanofi R&D, Framingham, MA, USA | Catherine.O'Riordan@sanofi.com | **Clinical Task Forces (FinnGen phase 3)** | **Eye diseases Task Force** |
| Samuel Lessard | Translational Sciences, Sanofi R&D, Framingham, MA, USA | samuel.lessard@sanofi.com | **Clinical Task Forces (FinnGen phase 3)** | **Eye diseases Task Force** |
| Suzanne Jacobs | Translational Sciences, Sanofi R&D, Framingham, MA, USA | Suzanne.Jacobs@sanofi.com | **Clinical Task Forces (FinnGen phase 3)** | **Eye diseases Task Force** |
| Satu Koskela | Finnish Red Cross Blood Service / Finnish Hematology Registry and Clinical Biobank, Helsinki, Finland | satu.koskela@veripalvelu.fi | **Clinical Task Forces (FinnGen phase 3)** | **Immune mediated diseases Task Force** |
| Anne Kerola | Institute for Molecular Medicine Finland (FIMM), HiLIFE, University of Helsinki, Helsinki, Finland | anne.kerola@helsinki.fi | **Clinical Task Forces (FinnGen phase 3)** | **Immune mediated diseases Task Force** |
| Elisa Lahtela | Institute for Molecular Medicine Finland (FIMM), HiLIFE, University of Helsinki, Helsinki, Finland | laura.lahtela@helsinki.fi | **Clinical Task Forces (FinnGen phase 3)** | **Immune mediated diseases Task Force** |
| Helen Cooper | Institute for Molecular Medicine Finland (FIMM), HiLIFE, University of Helsinki, Helsinki, Finland | helen.cooper@helsinki.fi | **Clinical Task Forces (FinnGen phase 3)** | **Immune mediated diseases Task Force** |
| Johanna Paltta | Institute for Molecular Medicine Finland (FIMM), HiLIFE, University of Helsinki, Helsinki, Finland; University of Turku, Turku, Finland | johanna.paltta@varha.fi | **Clinical Task Forces (FinnGen phase 3)** | **Immune mediated diseases Task Force** |
| Jukka Koskela | Institute for Molecular Medicine Finland (FIMM), HiLIFE, University of Helsinki, Helsinki, Finland | jukka.koskela@helsinki.fi | **Clinical Task Forces (FinnGen phase 3)** | **Immune mediated diseases Task Force** |
| Mark Daly | Institute for Molecular Medicine Finland (FIMM), HiLIFE, University of Helsinki, Helsinki, Finland; Broad Institute of MIT and Harvard; Massachusetts General Hospital, Boston, MA, United States | mjdaly@broadinstitute.com | **Clinical Task Forces (FinnGen phase 3)** | **Immune mediated diseases Task Force** |
| Mary Pat Reeve | Institute for Molecular Medicine Finland (FIMM), HiLIFE, University of Helsinki, Helsinki, Finland; Broad Institute, Cambridge, MA, United States | mary.reeve@helsinki.fi | **Clinical Task Forces (FinnGen phase 3)** | **Immune mediated diseases Task Force** |
| Vincent Llorens | Institute for Molecular Medicine Finland (FIMM), HiLIFE, University of Helsinki, Helsinki, Finland | vincent.llorens@helsinki.fi | **Clinical Task Forces (FinnGen phase 3)** | **Immune mediated diseases Task Force** |
| Martti Färkkilä | Hospital District of Helsinki and Uusimaa, Helsinki, Finland | martti.farkkila@hus.fi | **Clinical Task Forces (FinnGen phase 3)** | **Immune mediated diseases Task Force** |
| Johannes Kettunen | Northern Finland Biobank Borealis / University of Oulu / Wellbeing services county of North Ostrobothnia, Oulu, Finland | johannes.Kettunen@oulu.fi | **Clinical Task Forces (FinnGen phase 3)** | **Immune mediated diseases Task Force** |
| Kaisa Tasanen-Maatta | Wellbeing services county of North Ostrobothnia, Oulu, Finland | kaisa.tasanen-maatta@oulu.fi | **Clinical Task Forces (FinnGen phase 3)** | **Immune mediated diseases Task Force** |
| Laura Huilaja | Wellbeing services county of North Ostrobothnia, Oulu, Finland | laura.huilaja@oulu.fi | **Clinical Task Forces (FinnGen phase 3)** | **Immune mediated diseases Task Force** |
| Minna Ruddock | Arctic biobank / University of Oulu, Oulu, Finland | minna.ruddock@oulu.fi | **Clinical Task Forces (FinnGen phase 3)** | **Immune mediated diseases Task Force** |
| Aki Havulinna | Finnish Institute for Health and Welfare (THL), Helsinki, Finland | aki.havulinna@thl.fi | **Clinical Task Forces (FinnGen phase 3)** | **Immune mediated diseases Task Force** |
| Antti Palomäki | University of Turku, Turku, Finland | ajpalo@utu.fi | **Clinical Task Forces (FinnGen phase 3)** | **Immune mediated diseases Task Force** |
| Laura Kuusalo | University of Turku, Turku, Finland | laanku@utu.fi | **Clinical Task Forces (FinnGen phase 3)** | **Immune mediated diseases Task Force** |
| Laura Pirilä | University of Turku, Turku, Finland | laura.pirila@fimnet.fi | **Clinical Task Forces (FinnGen phase 3)** | **Immune mediated diseases Task Force** |
| Fedik Rahimov | AbbVie, Chicago, IL, United States | fedik.rahimov@abbvie.com | **Clinical Task Forces (FinnGen phase 3)** | **Immune mediated diseases Task Force** |
| Jan Freudenberg | AbbVie, Chicago, IL, United States | jan.freudenberg@abbvie.com | **Clinical Task Forces (FinnGen phase 3)** | **Immune mediated diseases Task Force** |
| Nizar Smaoui | AbbVie, Chicago, IL, United States | nizar.smaoui@abbvie.com | **Clinical Task Forces (FinnGen phase 3)** | **Immune mediated diseases Task Force** |
| Bram Prins | Astra Zeneca, Cambridge, United Kingdom | bram.prins@astrazeneca.com | **Clinical Task Forces (FinnGen phase 3)** | **Immune mediated diseases Task Force** |
| Coralie Viollet | Astra Zeneca, Cambridge, United Kingdom | coralie.viollet@astrazeneca.com | **Clinical Task Forces (FinnGen phase 3)** | **Immune mediated diseases Task Force** |
| Eleanor Wheeler | Astra Zeneca, Cambridge, United Kingdom | eleanor.wheeler@astrazeneca.com | **Clinical Task Forces (FinnGen phase 3)** | **Immune mediated diseases Task Force** |
| Kousik Kundu | Astra Zeneca, Cambridge, United Kingdom | kousik.kundu@astrazeneca.com | **Clinical Task Forces (FinnGen phase 3)** | **Immune mediated diseases Task Force** |
| Santosh Atanur | Astra Zeneca, Cambridge, United Kingdom | santosh.atanur@astrazeneca.com | **Clinical Task Forces (FinnGen phase 3)** | **Immune mediated diseases Task Force** |
| Hans van Leeuwen | Bayer AG, Leverkusen, Germany | hans.vanleeuwen@bayer.com | **Clinical Task Forces (FinnGen phase 3)** | **Immune mediated diseases Task Force** |
| Himanshu Manchanda | Bayer AG, Leverkusen, Germany | himanshu.manchanda@bayer.com | **Clinical Task Forces (FinnGen phase 3)** | **Immune mediated diseases Task Force** |
| Karl Heilbron | Bayer AG, Leverkusen, Germany | karl.heilbron@bayer.com | **Clinical Task Forces (FinnGen phase 3)** | **Immune mediated diseases Task Force** |
| Martin Rao | Bayer AG, Leverkusen, Germany | martin.rao@bayer.com | **Clinical Task Forces (FinnGen phase 3)** | **Immune mediated diseases Task Force** |
| Nicole Schmidt | Bayer AG, Leverkusen, Germany | nicole.schmidt1@bayer.com | **Clinical Task Forces (FinnGen phase 3)** | **Immune mediated diseases Task Force** |
| Samu Kurki | Bayer AG, Leverkusen, Germany | samu.kurki@bayer.com | **Clinical Task Forces (FinnGen phase 3)** | **Immune mediated diseases Task Force** |
| Ellen Tsai | Biogen, Cambridge, MA, United States | ellen.tsai@biogen.com | **Clinical Task Forces (FinnGen phase 3)** | **Immune mediated diseases Task Force** |
| Ketian Yu | Biogen, Cambridge, MA, United States | ketian.yu@biogen.com | **Clinical Task Forces (FinnGen phase 3)** | **Immune mediated diseases Task Force** |
| Stephanie Loomis | Biogen, Cambridge, MA, United States | stephanie.loomis@biogen.com | **Clinical Task Forces (FinnGen phase 3)** | **Immune mediated diseases Task Force** |
| Benjamin Sun | Bristol Myers Squibb, New York, NY, United States | benjamin.sun@bms.com | **Clinical Task Forces (FinnGen phase 3)** | **Immune mediated diseases Task Force** |
| Cara Carty | Bristol Myers Squibb, New York, NY, United States | cara.carty@bms.com | **Clinical Task Forces (FinnGen phase 3)** | **Immune mediated diseases Task Force** |
| Emily Holzinger | Bristol Myers Squibb, New York, NY, United States | Emily.Holzinger@bms.com | **Clinical Task Forces (FinnGen phase 3)** | **Immune mediated diseases Task Force** |
| Michael Turchin | Bristol Myers Squibb, New York, NY, United States | Michael.turchin@bms.com | **Clinical Task Forces (FinnGen phase 3)** | **Immune mediated diseases Task Force** |
| Neelakshi Jog | Bristol Myers Squibb, New York, NY, United States | neelakshi.jog@bms.com | **Clinical Task Forces (FinnGen phase 3)** | **Immune mediated diseases Task Force** |
| Frank Li | Boehringer Ingelheim, Ingelheim am Rhein, Germany | frank.li@boehringer-ingelheim.com | **Clinical Task Forces (FinnGen phase 3)** | **Immune mediated diseases Task Force** |
| Zhihao Ding | Boehringer Ingelheim, Ingelheim am Rhein, Germany | zhihao.ding@boehringer-ingelheim.com | **Clinical Task Forces (FinnGen phase 3)** | **Immune mediated diseases Task Force** |
| Cameron Adams | Genentech, San Francisco, CA, United States | adams.cameron@gene.com | **Clinical Task Forces (FinnGen phase 3)** | **Immune mediated diseases Task Force** |
| Mark McCarthy | Genentech, San Francisco, CA, United States | mccarm10@gene.com | **Clinical Task Forces (FinnGen phase 3)** | **Immune mediated diseases Task Force** |
| Michael Rothenberg | Genentech, San Francisco, CA, United States | rothenm1@gene.com | **Clinical Task Forces (FinnGen phase 3)** | **Immune mediated diseases Task Force** |
| Rion Pendergrass | Genentech, San Francisco, CA, United States | pendergrass.rion@gene.com | **Clinical Task Forces (FinnGen phase 3)** | **Immune mediated diseases Task Force** |
| Diana L.Cousminer | GlaxoSmithKline, Collegeville, PA, United States | diana.l.cousminer@gsk.com | **Clinical Task Forces (FinnGen phase 3)** | **Immune mediated diseases Task Force** |
| Jagtar Nijjar | GlaxoSmithKline, Collegeville, PA, United States | jagtar.s.nijjar@gsk.com | **Clinical Task Forces (FinnGen phase 3)** | **Immune mediated diseases Task Force** |
| Jessica Chao | GlaxoSmithKline, Collegeville, PA, United States | jessica.x.chao@gsk.com | **Clinical Task Forces (FinnGen phase 3)** | **Immune mediated diseases Task Force** |
| Joanna C.Betts | GlaxoSmithKline, Collegeville, PA, United States | joanna.c.betts@gsk.com | **Clinical Task Forces (FinnGen phase 3)** | **Immune mediated diseases Task Force** |
| Jonathan M.Davitte | GlaxoSmithKline, Collegeville, PA, United States | jonathan.m.davitte@gsk.com | **Clinical Task Forces (FinnGen phase 3)** | **Immune mediated diseases Task Force** |
| Linda McGarthy | GlaxoSmithKline, Collegeville, PA, United States | linda.c.mccarthy@gsk.com | **Clinical Task Forces (FinnGen phase 3)** | **Immune mediated diseases Task Force** |
| Michal Magid | GlaxoSmithKline, Collegeville, PA, United States | michal.m.magid-slav@gsk.com | **Clinical Task Forces (FinnGen phase 3)** | **Immune mediated diseases Task Force** |
| Shashank Jariwala | GlaxoSmithKline, Collegeville, PA, United States | shashank.x.jariwala@gsk.com | **Clinical Task Forces (FinnGen phase 3)** | **Immune mediated diseases Task Force** |
| Dawn Waterworth | Johnson & Johnson Innovative Medicine, Spring House, PA, United States | dwaterwo@its.jnj.com | **Clinical Task Forces (FinnGen phase 3)** | **Immune mediated diseases Task Force** |
| Amy Hart | Johnson & Johnson Innovative Medicine, Spring House, PA, United States | ahart13@its.jnj.com | **Clinical Task Forces (FinnGen phase 3)** | **Immune mediated diseases Task Force** |
| Brice Keyes | Johnson & Johnson Innovative Medicine, Spring House, PA, United States | bkeyes1@its.jnj.com | **Clinical Task Forces (FinnGen phase 3)** | **Immune mediated diseases Task Force** |
| John Kwon | Johnson & Johnson Innovative Medicine, Spring House, PA, United States | JKwon10@ITS.JNJ.com | **Clinical Task Forces (FinnGen phase 3)** | **Immune mediated diseases Task Force** |
| Jonathan Sherlock | Johnson & Johnson Innovative Medicine, Spring House, PA, United States | JSherlo1@its.jnj.com | **Clinical Task Forces (FinnGen phase 3)** | **Immune mediated diseases Task Force** |
| Matt Loza | Johnson & Johnson Innovative Medicine, Spring House, PA, United States | MLoza@its.jnj.com | **Clinical Task Forces (FinnGen phase 3)** | **Immune mediated diseases Task Force** |
| Elisabeth Vollmann | Merck, Kenilworth, NJ, United States | elisabeth.vollmann@merck.com | **Clinical Task Forces (FinnGen phase 3)** | **Immune mediated diseases Task Force** |
| Jozsef Karman | Merck, Kenilworth, NJ, United States | jozsef.karman@merck.com | **Clinical Task Forces (FinnGen phase 3)** | **Immune mediated diseases Task Force** |
| Julie Fiore | Merck, Kenilworth, NJ, United States | julie.fiore1@merck.com | **Clinical Task Forces (FinnGen phase 3)** | **Immune mediated diseases Task Force** |
| Rajesh Kamath | Merck, Kenilworth, NJ, United States | rajesh.kamath@merck.com | **Clinical Task Forces (FinnGen phase 3)** | **Immune mediated diseases Task Force** |
| Enrico Ferrero | Novartis Institutes for BioMedical Research, Cambridge, MA, United States | enrico.ferrero@novartis.com | **Clinical Task Forces (FinnGen phase 3)** | **Immune mediated diseases Task Force** |
| Jonas Zierer | Novartis Institutes for BioMedical Research, Cambridge, MA, United States | jonas.zierer@novartis.com | **Clinical Task Forces (FinnGen phase 3)** | **Immune mediated diseases Task Force** |
| Nikos Patsopoulos | Novartis Institutes for BioMedical Research, Cambridge, MA, United States | nikos.patsopoulos@novartis.com | **Clinical Task Forces (FinnGen phase 3)** | **Immune mediated diseases Task Force** |
| Erin Macdonald-Dunlop | Pfizer, New York, NY, United States | erin.macdonald-dunlop@pfizer.com | **Clinical Task Forces (FinnGen phase 3)** | **Immune mediated diseases Task Force** |
| Jessica Chung | Pfizer, New York, NY, United States | jessica.chung@pfizer.com | **Clinical Task Forces (FinnGen phase 3)** | **Immune mediated diseases Task Force** |
| Michael McLean | Pfizer, New York, NY, United States | Michael.McLean@pfizer.com | **Clinical Task Forces (FinnGen phase 3)** | **Immune mediated diseases Task Force** |
| Hamid Mattoo | Translational Sciences, Sanofi R&D, Framingham, MA, USA | Hamid.Mattoo@sanofi.com | **Clinical Task Forces (FinnGen phase 3)** | **Immune mediated diseases Task Force** |
| Aarno Palotie | Institute for Molecular Medicine Finland (FIMM), HiLIFE, University of Helsinki, Helsinki, Finland; Broad Institute of MIT and Harvard; Massachusetts General Hospital, Boston, MA, United States | aarno.palotie@helsinki.fi | **Clinical Task Forces (FinnGen phase 3)** | **Kidney diseases Task Force** |
| Elisa Lahtela | Institute for Molecular Medicine Finland (FIMM), HiLIFE, University of Helsinki, Helsinki, Finland | laura.lahtela@helsinki.fi | **Clinical Task Forces (FinnGen phase 3)** | **Kidney diseases Task Force** |
| Helen Cooper | Institute for Molecular Medicine Finland (FIMM), HiLIFE, University of Helsinki, Helsinki, Finland | helen.cooper@helsinki.fi | **Clinical Task Forces (FinnGen phase 3)** | **Kidney diseases Task Force** |
| Jukka Koskela | Institute for Molecular Medicine Finland (FIMM), HiLIFE, University of Helsinki, Helsinki, Finland | jukka.koskela@helsinki.fi | **Clinical Task Forces (FinnGen phase 3)** | **Kidney diseases Task Force** |
| Mark Daly | Institute for Molecular Medicine Finland (FIMM), HiLIFE, University of Helsinki, Helsinki, Finland; Broad Institute of MIT and Harvard; Massachusetts General Hospital, Boston, MA, United States | mjdaly@broadinstitute.com | **Clinical Task Forces (FinnGen phase 3)** | **Kidney diseases Task Force** |
| Mary Pat Reeve | Institute for Molecular Medicine Finland (FIMM), HiLIFE, University of Helsinki, Helsinki, Finland; Broad Institute, Cambridge, MA, United States | mary.reeve@helsinki.fi | **Clinical Task Forces (FinnGen phase 3)** | **Kidney diseases Task Force** |
| Raymond Walters | Institute for Molecular Medicine Finland (FIMM), HiLIFE, University of Helsinki, Helsinki, Finland; Broad Institute, Cambridge, MA, United States | rwalters@broadinstitute.org | **Clinical Task Forces (FinnGen phase 3)** | **Kidney diseases Task Force** |
| Rodos Rodosthenous | Institute for Molecular Medicine Finland (FIMM), HiLIFE, University of Helsinki, Helsinki, Finland | rodos.rodosthenous@helsinki.fi | **Clinical Task Forces (FinnGen phase 3)** | **Kidney diseases Task Force** |
| Jouni Lauronen | Finnish Red Cross Blood Service / Finnish Hematology Registry and Clinical Biobank, Helsinki, Finland | jouni.lauronen@veripalvelu.fi | **Clinical Task Forces (FinnGen phase 3)** | **Kidney diseases Task Force** |
| Adrian Banerji | Institute for Molecular Medicine Finland (FIMM), HiLIFE, University of Helsinki, Helsinki, Finland; Broad Institute & Harvard Medical School, Cambridge, United States | adrian.banerji@childrens.harvard.edu | **Clinical Task Forces (FinnGen phase 3)** | **Kidney diseases Task Force** |
| Matthew Sampson | Broad Institute, Cambridge, MA, United States; Harvard Medical School, Cambridge, United States | matthew.sampson@childrens.harvard.edu | **Clinical Task Forces (FinnGen phase 3)** | **Kidney diseases Task Force** |
| Michelle McNulty | Institute for Molecular Medicine Finland (FIMM), HiLIFE, University of Helsinki, Helsinki, Finland; Broad Institute & Harvard Medical School, Cambridge, United States | michelle.mcnulty@childrens.harvard.edu | **Clinical Task Forces (FinnGen phase 3)** | **Kidney diseases Task Force** |
| Daniel Gordin | Helsinki University Hospital and University of Helsinki, Helsinki, Finland | daniel.gordin@helsinki.fi | **Clinical Task Forces (FinnGen phase 3)** | **Kidney diseases Task Force** |
| Patrik Finne | Helsinki University Hospital and University of Helsinki, Helsinki, Finland | patrik.finne@helsinki.fi | **Clinical Task Forces (FinnGen phase 3)** | **Kidney diseases Task Force** |
| Mika Kähönen | Finnish Clinical Biobank Tampere / University of Tampere / Wellbeing Services County of Pirkanmaa, Tampere, Finland | mika.kahonen@tuni.fi | **Clinical Task Forces (FinnGen phase 3)** | **Kidney diseases Task Force** |
| Tapio Hellman | University of Turku, Turku, Finland | tapio.hellman@tyks.fi | **Clinical Task Forces (FinnGen phase 3)** | **Kidney diseases Task Force** |
| Teemu Niiranen | University of Turku, Turku, Finland; Finnish Institute for Health and Welfare (THL), Helsinki, Finland | tejuni@utu.fi | **Clinical Task Forces (FinnGen phase 3)** | **Kidney diseases Task Force** |
| Dirk Paul | Astra Zeneca, Cambridge, United Kingdom | dirk.paul@astrazeneca.com | **Clinical Task Forces (FinnGen phase 3)** | **Kidney diseases Task Force** |
| Ioanna Tachmazidou | Astra Zeneca, Cambridge, United Kingdom | ioanna.tachmazidou@astrazeneca.com | **Clinical Task Forces (FinnGen phase 3)** | **Kidney diseases Task Force** |
| Hans van Leeuwen | Bayer AG, Leverkusen, Germany | hans.vanleeuwen@bayer.com | **Clinical Task Forces (FinnGen phase 3)** | **Kidney diseases Task Force** |
| Johanna Mielke | Bayer AG, Leverkusen, Germany | johanna.mielke@bayer.com | **Clinical Task Forces (FinnGen phase 3)** | **Kidney diseases Task Force** |
| Juho Immonen | Bayer AG, Leverkusen, Germany | juho.immonen@bayer.com | **Clinical Task Forces (FinnGen phase 3)** | **Kidney diseases Task Force** |
| Thomas Battram | Bayer AG, Leverkusen, Germany | thomas.battram@bayer.com | **Clinical Task Forces (FinnGen phase 3)** | **Kidney diseases Task Force** |
| Tobias Hogrebe | Bayer AG, Leverkusen, Germany | tobias.hogrebe@bayer.com | **Clinical Task Forces (FinnGen phase 3)** | **Kidney diseases Task Force** |
| Ketian Yu | Biogen, Cambridge, MA, United States | ketian.yu@biogen.com | **Clinical Task Forces (FinnGen phase 3)** | **Kidney diseases Task Force** |
| Benjamin Sun | Bristol Myers Squibb, New York, NY, United States | Benjamin.Sun@bms.com | **Clinical Task Forces (FinnGen phase 3)** | **Kidney diseases Task Force** |
| Janie Shelton | Bristol Myers Squibb, New York, NY, United States | janie.shelton@bms.com | **Clinical Task Forces (FinnGen phase 3)** | **Kidney diseases Task Force** |
| Yao Hu | Boehringer Ingelheim, Ingelheim am Rhein, Germany | yao.hu@boehringer-ingelheim.com | **Clinical Task Forces (FinnGen phase 3)** | **Kidney diseases Task Force** |
| Zhihao Ding | Boehringer Ingelheim, Ingelheim am Rhein, Germany | zhihao.ding@boehringer-ingelheim.com | **Clinical Task Forces (FinnGen phase 3)** | **Kidney diseases Task Force** |
| Rion Pendergrass | Genentech, San Francisco, CA, United States | pendergrass.rion@gene.com | **Clinical Task Forces (FinnGen phase 3)** | **Kidney diseases Task Force** |
| Sergio Dellepiane | Genentech, San Francisco, CA, United States | dellepis@gene.com | **Clinical Task Forces (FinnGen phase 3)** | **Kidney diseases Task Force** |
| Audrey Chu | GlaxoSmithKline, Collegeville, PA, United States | audrey.y.chu@gsk.com | **Clinical Task Forces (FinnGen phase 3)** | **Kidney diseases Task Force** |
| Chris Floyd | GlaxoSmithKline, Collegeville, PA, United States | christopher.n.floyd@gsk.com | **Clinical Task Forces (FinnGen phase 3)** | **Kidney diseases Task Force** |
| Dan Swerdlow | GlaxoSmithKline, Collegeville, PA, United States | daniel.8.swerdlow@gsk.com | **Clinical Task Forces (FinnGen phase 3)** | **Kidney diseases Task Force** |
| Erding Hu | GlaxoSmithKline, Collegeville, PA, United States | erding.hu@gsk.com | **Clinical Task Forces (FinnGen phase 3)** | **Kidney diseases Task Force** |
| Jonathan Davitte | GlaxoSmithKline, Collegeville, PA, United States | jonathan.m.davitte@gsk.com | **Clinical Task Forces (FinnGen phase 3)** | **Kidney diseases Task Force** |
| Prerak Desai | GlaxoSmithKline, Collegeville, PA, United States | prerak.x.desai@gsk.com | **Clinical Task Forces (FinnGen phase 3)** | **Kidney diseases Task Force** |
| Stephen Haddad | GlaxoSmithKline, Collegeville, PA, United States | stephen.a.haddad@gsk.com | **Clinical Task Forces (FinnGen phase 3)** | **Kidney diseases Task Force** |
| Dermot Reilly | Johnson & Johnson Innovative Medicine, Spring House, PA, United States | DReill11@its.jnj.com | **Clinical Task Forces (FinnGen phase 3)** | **Kidney diseases Task Force** |
| P. Dunnmon | Johnson & Johnson Innovative Medicine, Spring House, PA, United States | PDunnmon@ITS.JNJ.com | **Clinical Task Forces (FinnGen phase 3)** | **Kidney diseases Task Force** |
| Karol Estrada | Maze Therapeutics, San Francisco, CA, United States | kestrada@mazetx.com | **Clinical Task Forces (FinnGen phase 3)** | **Kidney diseases Task Force** |
| Rob Graham | Maze Therapeutics, San Francisco, CA, United States | rgraham@mazetx.com | **Clinical Task Forces (FinnGen phase 3)** | **Kidney diseases Task Force** |
| Sahar Mozzafari | Maze Therapeutics, San Francisco, CA, United States | smozaffari@mazetx.com | **Clinical Task Forces (FinnGen phase 3)** | **Kidney diseases Task Force** |
| Nancy Finkel | Novartis Institutes for BioMedical Research, Cambridge, MA, United States | nancy.finkel@novartis.com | **Clinical Task Forces (FinnGen phase 3)** | **Kidney diseases Task Force** |
| Sabina Pfister | Novartis Institutes for BioMedical Research, Cambridge, MA, United States | sabina.pfister@novartis.com | **Clinical Task Forces (FinnGen phase 3)** | **Kidney diseases Task Force** |
| Shola Richards | Novartis Institutes for BioMedical Research, Cambridge, MA, United States | shola.richards@novartis.com | **Clinical Task Forces (FinnGen phase 3)** | **Kidney diseases Task Force** |
| Joshua Chiou | Pfizer, New York, NY, United States | joshua.chiou@pfizer.com | **Clinical Task Forces (FinnGen phase 3)** | **Kidney diseases Task Force** |
| Ying Wu | Pfizer, New York, NY, United States | ying.wu3@pfizer.com | **Clinical Task Forces (FinnGen phase 3)** | **Kidney diseases Task Force** |
| Katherine Klinger | Translational Sciences, Sanofi R&D, Framingham, MA, USA | katherine.klinger@sanofi.com | **Clinical Task Forces (FinnGen phase 3)** | **Kidney diseases Task Force** |
| Matti Vuori | University of Turku, Turku, Finland | makvuo@utu.fi | **Clinical Task Forces (FinnGen phase 3)** | **Metabolic diseases Task Force** |
| Teemu Niiranen | University of Turku, Turku, Finland; Finnish Institute for Health and Welfare (THL), Helsinki, Finland | tejuni@utu.fi | **Clinical Task Forces (FinnGen phase 3)** | **Metabolic diseases Task Force** |
| Bridget Riley-Gillis | AbbVie, Chicago, IL, United States | bridget.rileygillis@abbvie.com | **Clinical Task Forces (FinnGen phase 3)** | **Metabolic diseases Task Force** |
| Nizar Smaoui | AbbVie, Chicago, IL, United States | nizar.smaoui@abbvie.com | **Clinical Task Forces (FinnGen phase 3)** | **Metabolic diseases Task Force** |
| Alix Berton | Bayer AG, Leverkusen, Germany | alix.berton@bayer.com | **Clinical Task Forces (FinnGen phase 3)** | **Metabolic diseases Task Force** |
| Hans van Leeuwen | Bayer AG, Leverkusen, Germany | hans.vanleeuwen@bayer.com | **Clinical Task Forces (FinnGen phase 3)** | **Metabolic diseases Task Force** |
| Chen Li | Bristol Myers Squibb, New York, NY, United States | Chen.Li@bms.com | **Clinical Task Forces (FinnGen phase 3)** | **Metabolic diseases Task Force** |
| Emily Holzinger | Bristol Myers Squibb, New York, NY, United States | Emily.Holzinger@bms.com | **Clinical Task Forces (FinnGen phase 3)** | **Metabolic diseases Task Force** |
| Anubha Mahajan | Genentech, San Francisco, CA, United States | mahajan.anubha@gene.com | **Clinical Task Forces (FinnGen phase 3)** | **Metabolic diseases Task Force** |
| Mark Mccarthy | Genentech, San Francisco, CA, United States | mccarthy.mark@gene.com | **Clinical Task Forces (FinnGen phase 3)** | **Metabolic diseases Task Force** |
| Christopher Deboever | Maze Therapeutics, San Francisco, CA, United States | cdeboever@mazetx.com | **Clinical Task Forces (FinnGen phase 3)** | **Metabolic diseases Task Force** |
| Karol Estrada | Maze Therapeutics, San Francisco, CA, United States | kestrada@mazetx.com | **Clinical Task Forces (FinnGen phase 3)** | **Metabolic diseases Task Force** |
| Robert Graham | Maze Therapeutics, San Francisco, CA, United States | rgraham@mazetx.com | **Clinical Task Forces (FinnGen phase 3)** | **Metabolic diseases Task Force** |
| Hye In Kim | Pfizer, New York, NY, United States | HyeIn.Kim@pfizer.com | **Clinical Task Forces (FinnGen phase 3)** | **Metabolic diseases Task Force** |
| Sivakumar Pitchumani | Pfizer, New York, NY, United States | Pitchumani.Sivakumar@pfizer.com | **Clinical Task Forces (FinnGen phase 3)** | **Metabolic diseases Task Force** |
| Sumedha Jassal | Pfizer, New York, NY, United States | Sumedha.Jassal@pfizer.com | **Clinical Task Forces (FinnGen phase 3)** | **Metabolic diseases Task Force** |
| Åsa Hedman | Pfizer, New York, NY, United States | asa.Hedman@pfizer.com | **Clinical Task Forces (FinnGen phase 3)** | **Metabolic diseases Task Force** |
| Aarno Palotie | Institute for Molecular Medicine Finland (FIMM), HiLIFE, University of Helsinki, Helsinki, Finland; Broad Institute of MIT and Harvard; Massachusetts General Hospital, Boston, MA, United States | aarno.palotie@helsinki.fi | **Clinical Task Forces (FinnGen phase 3)** | **Neurodegenerative diseases Task Force** |
| Austin Argentieri | Institute for Molecular Medicine Finland (FIMM), HiLIFE, University of Helsinki, Helsinki, Finland; Broad Institute, Cambridge, MA, United States | aargentieri@mgh.harvard.edu | **Clinical Task Forces (FinnGen phase 3)** | **Neurodegenerative diseases Task Force** |
| Aoxing Liu | Institute for Molecular Medicine Finland (FIMM), HiLIFE, University of Helsinki, Helsinki, Finland | aoxing.liu@helsinki.fi | **Clinical Task Forces (FinnGen phase 3)** | **Neurodegenerative diseases Task Force** |
| Eero Vuoksimaa | Institute for Molecular Medicine Finland (FIMM), HiLIFE, University of Helsinki, Helsinki, Finland | eero.vuoksimaa@helsinki.fi | **Clinical Task Forces (FinnGen phase 3)** | **Neurodegenerative diseases Task Force** |
| Elisa Lahtela | Institute for Molecular Medicine Finland (FIMM), HiLIFE, University of Helsinki, Helsinki, Finland | laura.lahtela@helsinki.fi | **Clinical Task Forces (FinnGen phase 3)** | **Neurodegenerative diseases Task Force** |
| Joni Lindbohm | Institute for Molecular Medicine Finland (FIMM), HiLIFE, University of Helsinki, Helsinki, Finland | joni.lindbohm@helsinki.fi | **Clinical Task Forces (FinnGen phase 3)** | **Neurodegenerative diseases Task Force** |
| Mark Daly | Institute for Molecular Medicine Finland (FIMM), HiLIFE, University of Helsinki, Helsinki, Finland; Broad Institute of MIT and Harvard; Massachusetts General Hospital, Boston, MA, United States | mjdaly@broadinstitute.com | **Clinical Task Forces (FinnGen phase 3)** | **Neurodegenerative diseases Task Force** |
| Mary Pat Reeve | Institute for Molecular Medicine Finland (FIMM), HiLIFE, University of Helsinki, Helsinki, Finland; Broad Institute, Cambridge, MA, United States | mary.reeve@helsinki.fi | **Clinical Task Forces (FinnGen phase 3)** | **Neurodegenerative diseases Task Force** |
| Paavo Häppölä | Institute for Molecular Medicine Finland (FIMM), HiLIFE, University of Helsinki, Helsinki, Finland | paavo.happola@helsinki.fi | **Clinical Task Forces (FinnGen phase 3)** | **Neurodegenerative diseases Task Force** |
| Zhiyu Yang | Institute for Molecular Medicine Finland (FIMM), HiLIFE, University of Helsinki, Helsinki, Finland | zhiyu.yang@helsinki.fi | **Clinical Task Forces (FinnGen phase 3)** | **Neurodegenerative diseases Task Force** |
| Eino Solje | University of Eastern Finland, Kuopio, Finland | eino.solje@uef.fi | **Clinical Task Forces (FinnGen phase 3)** | **Neurodegenerative diseases Task Force** |
| Mikko Hiltunen | University of Eastern Finland, Kuopio, Finland | mikko.hiltunen@uef.fi | **Clinical Task Forces (FinnGen phase 3)** | **Neurodegenerative diseases Task Force** |
| Valtteri Julkunen | University of Eastern Finland and Kuopio University Hospital, Kuopio, Finland | Valtteri.Julkunen@pshyvinvointialue.fi | **Clinical Task Forces (FinnGen phase 3)** | **Neurodegenerative diseases Task Force** |
| Ville Leinonen | University of Eastern Finland and Kuopio University Hospital, Kuopio, Finland | ville.leinonen@pshyvinvointialue.fi | **Clinical Task Forces (FinnGen phase 3)** | **Neurodegenerative diseases Task Force** |
| Hanna Kujala | Biobank of Eastern Finland / University of Eastern Finland / Wellbeing services county of North Savo, Kuopio, Finland | Hanna.Kujala@pshyvinvointialue.fi | **Clinical Task Forces (FinnGen phase 3)** | **Neurodegenerative diseases Task Force** |
| Aki Havulinna | Finnish Institute for Health and Welfare (THL), Helsinki, Finland | aki.havulinna@thl.fi | **Clinical Task Forces (FinnGen phase 3)** | **Neurodegenerative diseases Task Force** |
| Roosa Kallionpää | University of Turku, Turku, Finland | roosa.kallionpaa@tyks.fi | **Clinical Task Forces (FinnGen phase 3)** | **Neurodegenerative diseases Task Force** |
| Minttu Marttila | University of Helsinki, Helsinki, Finland | minttu.marttila@helsinki.fi | **Clinical Task Forces (FinnGen phase 3)** | **Neurodegenerative diseases Task Force** |
| Britney Milkovich | AbbVie, Chicago, IL, United States | britney.milkovich@abbvie.com | **Clinical Task Forces (FinnGen phase 3)** | **Neurodegenerative diseases Task Force** |
| Jan Freudenberg | AbbVie, Chicago, IL, United States | jan.freudenberg@abbvie.com | **Clinical Task Forces (FinnGen phase 3)** | **Neurodegenerative diseases Task Force** |
| Andrew Lowe | Astra Zeneca, Cambridge, United Kingdom | andrew.lowe@astrazeneca.com | **Clinical Task Forces (FinnGen phase 3)** | **Neurodegenerative diseases Task Force** |
| Ioanna Tachmazidou | Astra Zeneca, Cambridge, United Kingdom | ioanna.tachmazidou@astrazeneca.com | **Clinical Task Forces (FinnGen phase 3)** | **Neurodegenerative diseases Task Force** |
| Thomas Spargo | Astra Zeneca, Cambridge, United Kingdom | thomas.spargo@astrazeneca.com | **Clinical Task Forces (FinnGen phase 3)** | **Neurodegenerative diseases Task Force** |
| Kritika Singh | Bristol Myers Squibb, New York, NY, United States | Kritika.Singh3@bms.com | **Clinical Task Forces (FinnGen phase 3)** | **Neurodegenerative diseases Task Force** |
| Peng Jiang | Bristol Myers Squibb, New York, NY, United States | Peng.Jiang@bms.com | **Clinical Task Forces (FinnGen phase 3)** | **Neurodegenerative diseases Task Force** |
| Stephanie Loomis | Bristol Myers Squibb, New York, NY, United States | stephanie.loomis@bms.com | **Clinical Task Forces (FinnGen phase 3)** | **Neurodegenerative diseases Task Force** |
| Anubha Mahajan | Genentech, San Francisco, CA, United States | mahaja11@gene.com | **Clinical Task Forces (FinnGen phase 3)** | **Neurodegenerative diseases Task Force** |
| Rion Pendergrass | Genentech, San Francisco, CA, United States | penders2@gene.com | **Clinical Task Forces (FinnGen phase 3)** | **Neurodegenerative diseases Task Force** |
| Damien Croteau-Chonka | GlaxoSmithKline, Collegeville, PA, United States | damien.c.croteau-chonka@gsk.com | **Clinical Task Forces (FinnGen phase 3)** | **Neurodegenerative diseases Task Force** |
| John Eicher | GlaxoSmithKline, Collegeville, PA, United States | john.d.eicher@gsk.com | **Clinical Task Forces (FinnGen phase 3)** | **Neurodegenerative diseases Task Force** |
| Prerak Desai | GlaxoSmithKline, Collegeville, PA, United States | prerak.x.desai@gsk.com | **Clinical Task Forces (FinnGen phase 3)** | **Neurodegenerative diseases Task Force** |
| Chris Whelan | Johnson & Johnson Innovative Medicine, Spring House, PA, United States | CWhelan4@ITS.JNJ.com | **Clinical Task Forces (FinnGen phase 3)** | **Neurodegenerative diseases Task Force** |
| Karen He | Johnson & Johnson Innovative Medicine, Spring House, PA, United States | KHe2@ITS.JNJ.com | **Clinical Task Forces (FinnGen phase 3)** | **Neurodegenerative diseases Task Force** |
| Qingqin Li | Johnson & Johnson Innovative Medicine, Spring House, PA, United States | qli2@its.jnj.com | **Clinical Task Forces (FinnGen phase 3)** | **Neurodegenerative diseases Task Force** |
| W Galpern | Johnson & Johnson Innovative Medicine, Spring House, PA, United States | wgalpern@ITS.JNJ.com | **Clinical Task Forces (FinnGen phase 3)** | **Neurodegenerative diseases Task Force** |
| Yanfei Zhang | Johnson & Johnson Innovative Medicine, Spring House, PA, United States | yzhan378@its.jnj.com | **Clinical Task Forces (FinnGen phase 3)** | **Neurodegenerative diseases Task Force** |
| Andrei Popescu | Merck, Kenilworth, NJ, United States | andrei.popescu@merck.com | **Clinical Task Forces (FinnGen phase 3)** | **Neurodegenerative diseases Task Force** |
| Delphine Fagegaltier | Merck, Kenilworth, NJ, United States | delphine.fagegaltier@merck.com | **Clinical Task Forces (FinnGen phase 3)** | **Neurodegenerative diseases Task Force** |
| Mari Niemi | Novartis Institutes for BioMedical Research, Cambridge, MA, United States | Mari.niemi@novartis.com | **Clinical Task Forces (FinnGen phase 3)** | **Neurodegenerative diseases Task Force** |
| Nikos Patsopoulos | Novartis Institutes for BioMedical Research, Cambridge, MA, United States | nikos.patsopoulos@novartis.com | **Clinical Task Forces (FinnGen phase 3)** | **Neurodegenerative diseases Task Force** |
| Katherine Klinger | Translational Sciences, Sanofi R&D, Framingham, MA, USA | katherine.klinger@sanofi.com | **Clinical Task Forces (FinnGen phase 3)** | **Neurodegenerative diseases Task Force** |
| Aarno Palotie | Institute for Molecular Medicine Finland (FIMM), HiLIFE, University of Helsinki, Helsinki, Finland; Broad Institute of MIT and Harvard; Massachusetts General Hospital, Boston, MA, United States | aarno.palotie@helsinki.fi | **Clinical Task Forces (FinnGen phase 3)** | **Pulmonology Task Force and fibrotic diseases interest group** |
| Elisa Lahtela | Institute for Molecular Medicine Finland (FIMM), HiLIFE, University of Helsinki, Helsinki, Finland | laura.lahtela@helsinki.fi | **Clinical Task Forces (FinnGen phase 3)** | **Pulmonology Task Force and fibrotic diseases interest group** |
| Jukka Koskela | Institute for Molecular Medicine Finland (FIMM), HiLIFE, University of Helsinki, Helsinki, Finland | jukka.koskela@helsinki.fi | **Clinical Task Forces (FinnGen phase 3)** | **Pulmonology Task Force and fibrotic diseases interest group** |
| Mark Daly | Institute for Molecular Medicine Finland (FIMM), HiLIFE, University of Helsinki, Helsinki, Finland; Broad Institute of MIT and Harvard; Massachusetts General Hospital, Boston, MA, United States | mjdaly@broadinstitute.org | **Clinical Task Forces (FinnGen phase 3)** | **Pulmonology Task Force and fibrotic diseases interest group** |
| Sanni Ruotsalainen | Institute for Molecular Medicine Finland (FIMM), HiLIFE, University of Helsinki, Helsinki, Finland | sanni.ruotsalainen@helsinki.fi | **Clinical Task Forces (FinnGen phase 3)** | **Pulmonology Task Force and fibrotic diseases interest group** |
| Susanna Lemmelä | Institute for Molecular Medicine Finland (FIMM), HiLIFE, University of Helsinki, Helsinki, Finland | susanna.lemmela@helsinki.fi | **Clinical Task Forces (FinnGen phase 3)** | **Pulmonology Task Force and fibrotic diseases interest group** |
| Tarja Laitinen | Institute for Molecular Medicine Finland (FIMM), HiLIFE, University of Helsinki, Helsinki, Finland | tarja.laitinen@helsinki.fi | **Clinical Task Forces (FinnGen phase 3)** | **Pulmonology Task Force and fibrotic diseases interest group** |
| Salla Ranta | Hospital District of Helsinki and Uusimaa, Helsinki, Finland | salla.a.ranta@hus.fi | **Clinical Task Forces (FinnGen phase 3)** | **Pulmonology Task Force and fibrotic diseases interest group** |
| Paavo Häppölä | Institute for Molecular Medicine Finland (FIMM), HiLIFE, University of Helsinki, Helsinki, Finland | paavo.happola@helsinki.fi | **Clinical Task Forces (FinnGen phase 3)** | **Pulmonology Task Force and fibrotic diseases interest group** |
| Paula Kauppi | Hospital District of Helsinki and Uusimaa, Helsinki, Finland | paula.kauppi@hus.fi | **Clinical Task Forces (FinnGen phase 3)** | **Pulmonology Task Force and fibrotic diseases interest group** |
| Tiinamaija Tuomi | Institute for Molecular Medicine Finland (FIMM), HiLIFE, University of Helsinki, Helsinki, Finland; Hospital District of Helsinki and Uusimaa, Helsinki, Finland | tiinamaija.tuomi@hus.fi | **Clinical Task Forces (FinnGen phase 3)** | **Pulmonology Task Force and fibrotic diseases interest group** |
| Raisa Serpi | Northern Finland Biobank Borealis / University of Oulu / Wellbeing services county of North Ostrobothnia, Oulu, Finland | Raisa.Serpi@ppshp.fi | **Clinical Task Forces (FinnGen phase 3)** | **Pulmonology Task Force and fibrotic diseases interest group** |
| Riitta Kaarteenaho | University of Oulu, Oulu, Finland | riitta.kaarteenaho@oulu.fi | **Clinical Task Forces (FinnGen phase 3)** | **Pulmonology Task Force and fibrotic diseases interest group** |
| Hannu Kankaanranta | University of Gothenburg, Gothenburg, Sweden/ Seinäjoki Central Hospital, Seinäjoki, Finland/ Tampere University, Tampere, Finland | hannu.kankaanranta@tuni.fi | **Clinical Task Forces (FinnGen phase 3)** | **Pulmonology Task Force and fibrotic diseases interest group** |
| Coralie Viollet | Astra Zeneca, Cambridge, United Kingdom | coralie.viollet@astrazeneca.com | **Clinical Task Forces (FinnGen phase 3)** | **Pulmonology Task Force and fibrotic diseases interest group** |
| Eleanor Wheeler | Astra Zeneca, Cambridge, United Kingdom | eleanor.wheeler@astrazeneca.com | **Clinical Task Forces (FinnGen phase 3)** | **Pulmonology Task Force and fibrotic diseases interest group** |
| Oliver Burren | Astra Zeneca, Cambridge, United Kingdom | oliver.burren@astrazeneca.com | **Clinical Task Forces (FinnGen phase 3)** | **Pulmonology Task Force and fibrotic diseases interest group** |
| Christoph Ogris | Boehringer Ingelheim, Ingelheim am Rhein, Germany | christoph.ogris@boehringer-ingelheim.com | **Clinical Task Forces (FinnGen phase 3)** | **Pulmonology Task Force and fibrotic diseases interest group** |
| Eric Simon | Boehringer Ingelheim, Ingelheim am Rhein, Germany | eric.simon@boehringer-ingelheim.com | **Clinical Task Forces (FinnGen phase 3)** | **Pulmonology Task Force and fibrotic diseases interest group** |
| Frank LI | Boehringer Ingelheim, Ingelheim am Rhein, Germany | frank.li@boehringer-ingelheim.com | **Clinical Task Forces (FinnGen phase 3)** | **Pulmonology Task Force and fibrotic diseases interest group** |
| Julio Cesar Bolivar Lopez | Boehringer Ingelheim, Ingelheim am Rhein, Germany | julio_cesar.bolivar_lopez@boehringer-ingelheim.com | **Clinical Task Forces (FinnGen phase 3)** | **Pulmonology Task Force and fibrotic diseases interest group** |
| Yao Hu | Boehringer Ingelheim, Ingelheim am Rhein, Germany | yao.hu@boehringer-ingelheim.com | **Clinical Task Forces (FinnGen phase 3)** | **Pulmonology Task Force and fibrotic diseases interest group** |
| Zhihao Ding | Boehringer Ingelheim, Ingelheim am Rhein, Germany | zhihao.ding@boehringer-ingelheim.com | **Clinical Task Forces (FinnGen phase 3)** | **Pulmonology Task Force and fibrotic diseases interest group** |
| Elena Sanchez | Bristol Myers Squibb, New York, NY, United States | elena.sanchez2@bms.com | **Clinical Task Forces (FinnGen phase 3)** | **Pulmonology Task Force and fibrotic diseases interest group** |
| Emily Holzinger | Bristol Myers Squibb, New York, NY, United States | Emily.Holzinger@bms.com | **Clinical Task Forces (FinnGen phase 3)** | **Pulmonology Task Force and fibrotic diseases interest group** |
| Joe Maranville | Bristol Myers Squibb, New York, NY, United States | joseph.maranville@bms.com | **Clinical Task Forces (FinnGen phase 3)** | **Pulmonology Task Force and fibrotic diseases interest group** |
| Lilith Moss | Bristol Myers Squibb, New York, NY, United States | Lilith.moss@bms.com | **Clinical Task Forces (FinnGen phase 3)** | **Pulmonology Task Force and fibrotic diseases interest group** |
| Michael Turchin | Bristol Myers Squibb, New York, NY, United States | michael.turchin@bms.com | **Clinical Task Forces (FinnGen phase 3)** | **Pulmonology Task Force and fibrotic diseases interest group** |
| Zijie Zhao | Bristol Myers Squibb, New York, NY, United States | Zijie.Zhao@bms.com | **Clinical Task Forces (FinnGen phase 3)** | **Pulmonology Task Force and fibrotic diseases interest group** |
| Diana Chang | Genentech, San Francisco, CA, United States | chang.diana@gene.com | **Clinical Task Forces (FinnGen phase 3)** | **Pulmonology Task Force and fibrotic diseases interest group** |
| Audrey Chu | GlaxoSmithKline, Collegeville, PA, United States | audrey.y.chu@gsk.com | **Clinical Task Forces (FinnGen phase 3)** | **Pulmonology Task Force and fibrotic diseases interest group** |
| Billy Fahy | GlaxoSmithKline, Collegeville, PA, United States | william.a.fahy@gsk.com | **Clinical Task Forces (FinnGen phase 3)** | **Pulmonology Task Force and fibrotic diseases interest group** |
| Jessica Chao | GlaxoSmithKline, Collegeville, PA, United States | jessica.x.chao@gsk.com | **Clinical Task Forces (FinnGen phase 3)** | **Pulmonology Task Force and fibrotic diseases interest group** |
| Joanna Betts | GlaxoSmithKline, Collegeville, PA, United States | joanna.c.betts@gsk.com | **Clinical Task Forces (FinnGen phase 3)** | **Pulmonology Task Force and fibrotic diseases interest group** |
| Jonathan Davitte | GlaxoSmithKline, Collegeville, PA, United States | jonathan.m.davitte@gsk.com | **Clinical Task Forces (FinnGen phase 3)** | **Pulmonology Task Force and fibrotic diseases interest group** |
| Paola Bronson | GlaxoSmithKline, Collegeville, PA, United States | paola.g.bronson@gsk.com | **Clinical Task Forces (FinnGen phase 3)** | **Pulmonology Task Force and fibrotic diseases interest group** |
| Prerak Desai | GlaxoSmithKline, Collegeville, PA, United States | prerak.x.desai@gsk.com | **Clinical Task Forces (FinnGen phase 3)** | **Pulmonology Task Force and fibrotic diseases interest group** |
| Dermot Reilly | Johnson & Johnson Innovative Medicine, Spring House, PA, United States | DReill11@its.jnj.com | **Clinical Task Forces (FinnGen phase 3)** | **Pulmonology Task Force and fibrotic diseases interest group** |
| Mona Selej | Johnson & Johnson Innovative Medicine, Spring House, PA, United States | mselej@its.jnj.com | **Clinical Task Forces (FinnGen phase 3)** | **Pulmonology Task Force and fibrotic diseases interest group** |
| P Dunnmon | Johnson & Johnson Innovative Medicine, Spring House, PA, United States | PDunnmon@ITS.JNJ.com | **Clinical Task Forces (FinnGen phase 3)** | **Pulmonology Task Force and fibrotic diseases interest group** |
| Jorge Del-aguila | Merck, Kenilworth, NJ, United States | jorge.del-aguila@merck.com | **Clinical Task Forces (FinnGen phase 3)** | **Pulmonology Task Force and fibrotic diseases interest group** |
| Jozsef Karman | Merck, Kenilworth, NJ, United States | jozsef.karman@merck.com | **Clinical Task Forces (FinnGen phase 3)** | **Pulmonology Task Force and fibrotic diseases interest group** |
| Travis Barr | Merck, Kenilworth, NJ, United States | travis.barr@merck.com | **Clinical Task Forces (FinnGen phase 3)** | **Pulmonology Task Force and fibrotic diseases interest group** |
| Katherine Mccauley | Novartis Institutes for BioMedical Research, Cambridge, MA, United States | katherine.mccauley@novartis.com | **Clinical Task Forces (FinnGen phase 3)** | **Pulmonology Task Force and fibrotic diseases interest group** |
| Xiaobo Xia | Novartis Institutes for BioMedical Research, Cambridge, MA, United States | xiaobo.xia@novartis.com | **Clinical Task Forces (FinnGen phase 3)** | **Pulmonology Task Force and fibrotic diseases interest group** |
| Madhurima Saxena | Pfizer, New York, NY, United States | Madhurima.Saxena@pfizer.com | **Clinical Task Forces (FinnGen phase 3)** | **Pulmonology Task Force and fibrotic diseases interest group** |
| Pitchumani Sivakumar | Pfizer, New York, NY, United States | Pitchumani.Sivakumar@pfizer.com | **Clinical Task Forces (FinnGen phase 3)** | **Pulmonology Task Force and fibrotic diseases interest group** |
| Sumedha Jassal | Pfizer, New York, NY, United States | sumedha.jassal@pfizer.com | **Clinical Task Forces (FinnGen phase 3)** | **Pulmonology Task Force and fibrotic diseases interest group** |
| David Habiel | Translational Sciences, Sanofi R&D, Framingham, MA, USA | david.habiel@sanofi.com | **Clinical Task Forces (FinnGen phase 3)** | **Pulmonology Task Force and fibrotic diseases interest group** |
| Guanling Huan | Translational Sciences, Sanofi R&D, Framingham, MA, USA | guanling.huang@sanofi.com | **Clinical Task Forces (FinnGen phase 3)** | **Pulmonology Task Force and fibrotic diseases interest group** |
| Marika Kaakinen | Institute for Molecular Medicine Finland (FIMM), HiLIFE, University of Helsinki, Helsinki, Finland | marika.kaakinen@helsinki.fi | **Clinical Task Forces (FinnGen phase 3)** | **Parkinson´s disease Task Force** |
| Mary Pat Reeve | Institute for Molecular Medicine Finland (FIMM), HiLIFE, University of Helsinki, Helsinki, Finland; Broad Institute, Cambridge, MA, United States | mary.reeve@helsinki.fi | **Clinical Task Forces (FinnGen phase 3)** | **Parkinson´s disease Task Force** |
| Filip Scheperjans | Hospital District of Helsinki and Uusimaa, Helsinki, Finland | Filip.Scheperjans@hus.fi | **Clinical Task Forces (FinnGen phase 3)** | **Parkinson´s disease Task Force** |
| Andrew Blumenfeld | AbbVie, Chicago, IL, United States | andrew.blumenfeld@abbvie.com | **Clinical Task Forces (FinnGen phase 3)** | **Parkinson´s disease Task Force** |
| Britney Milkovich | AbbVie, Chicago, IL, United States | Britney.Milkovich@abbvie.com | **Clinical Task Forces (FinnGen phase 3)** | **Parkinson´s disease Task Force** |
| Jan Freudenberg | AbbVie, Chicago, IL, United States | Jan.Freudenberg@abbvie.com | **Clinical Task Forces (FinnGen phase 3)** | **Parkinson´s disease Task Force** |
| Tushar Kumar | AbbVie, Chicago, IL, United States | tushar.kumar@abbvie.com | **Clinical Task Forces (FinnGen phase 3)** | **Parkinson´s disease Task Force** |
| Hans van Leeuwen | Bayer AG, Leverkusen, Germany | hans.vanleeuwen@bayer.com | **Clinical Task Forces (FinnGen phase 3)** | **Parkinson´s disease Task Force** |
| Juho Immonen | Bayer AG, Leverkusen, Germany | juho.immonen@bayer.com | **Clinical Task Forces (FinnGen phase 3)** | **Parkinson´s disease Task Force** |
| Samu Kurki | Bayer AG, Leverkusen, Germany | samu.kurki@bayer.com | **Clinical Task Forces (FinnGen phase 3)** | **Parkinson´s disease Task Force** |
| Coro Paisan-Ruiz | Biogen, Cambridge, MA, United States | coro.paisanruiz@biogen.com | **Clinical Task Forces (FinnGen phase 3)** | **Parkinson´s disease Task Force** |
| Anna Podgornaia | Bristol Myers Squibb, New York, NY, United States | Anna.Podgornaia@bms.com | **Clinical Task Forces (FinnGen phase 3)** | **Parkinson´s disease Task Force** |
| Benjamin Sun | Bristol Myers Squibb, New York, NY, United States | Benjamin.Sun@bms.com | **Clinical Task Forces (FinnGen phase 3)** | **Parkinson´s disease Task Force** |
| Janie Shelton | Bristol Myers Squibb, New York, NY, United States | Janie.Shelton@bms.com | **Clinical Task Forces (FinnGen phase 3)** | **Parkinson´s disease Task Force** |
| Peng Jiang | Bristol Myers Squibb, New York, NY, United States | Peng.Jiang@bms.com | **Clinical Task Forces (FinnGen phase 3)** | **Parkinson´s disease Task Force** |
| Stephanie Loomis | Bristol Myers Squibb, New York, NY, United States | stephanie.loomis@bms.com | **Clinical Task Forces (FinnGen phase 3)** | **Parkinson´s disease Task Force** |
| Tushar Bhangale | Genentech, San Francisco, CA, United States | bhangale.tushar@gene.com | **Clinical Task Forces (FinnGen phase 3)** | **Parkinson´s disease Task Force** |
| John Eicher | GlaxoSmithKline, Collegeville, PA, United States | john.d.eicher@gsk.com | **Clinical Task Forces (FinnGen phase 3)** | **Parkinson´s disease Task Force** |
| Abolfazl Doostparast Torshizi | Johnson & Johnson Innovative Medicine, Spring House, PA, United States | ADoostpa@ITS.JNJ.com | **Clinical Task Forces (FinnGen phase 3)** | **Parkinson´s disease Task Force** |
| Aristide Merola | Merck, Kenilworth, NJ, United States | aristide.merola@merck.com | **Clinical Task Forces (FinnGen phase 3)** | **Parkinson´s disease Task Force** |
| Oliver Freeman | Merck, Kenilworth, NJ, United States | oliver.freeman@msd.com | **Clinical Task Forces (FinnGen phase 3)** | **Parkinson´s disease Task Force** |
| Simonne Longerich | Merck, Kenilworth, NJ, United States | simonne.longerich@merck.com | **Clinical Task Forces (FinnGen phase 3)** | **Parkinson´s disease Task Force** |
| Mari Niemi | Novartis Institutes for BioMedical Research, Cambridge, MA, United States | mari.niemi@novartis.com | **Clinical Task Forces (FinnGen phase 3)** | **Parkinson´s disease Task Force** |
| Katherine Klinger | Translational Sciences, Sanofi R&D, Framingham, MA, USA | Katherine.Klinger@sanofi.com | **Clinical Task Forces (FinnGen phase 3)** | **Parkinson´s disease Task Force** |
